# Genome-wide Genetic Mutations Accumulated in Pigs Genome-edited for Xenotransplantation and Their Filial Generation

**DOI:** 10.1093/gpbjnl/qzaf071

**Published:** 2025-08-20

**Authors:** Xueyun Huo, Xianhui Sun, Xiangyang Xing, Jing Lu, Jingjing Zhang, Yanyan Jiang, Xiao Zhu, Changlong Li, Jianyi Lv, Meng Guo, Lixue Cao, Xin Liu, Zhenwen Chen, Dengke Pan, Shunmin He, Chen Zhang, Xiaoyan Du

**Affiliations:** School of Basic Medical Sciences, Capital Medical University, Beijing 100069, China; Laboratory for Clinical Medicine, Capital Medical University, Beijing 100069, China; Beijing Key Laboratory of Cancer Invasion and Metastasis Research, Beijing 100069, China; Key Laboratory of Epigenetic Regulation and Intervention, Institute of Biophysics, Chinese Academy of Sciences, Beijing 100101, China; University of Chinese Academy of Sciences, Beijing 100049, China; Chengdu ClonOrgan Biotechnology Co., Ltd., Chengdu 610041, China; School of Basic Medical Sciences, Capital Medical University, Beijing 100069, China; Laboratory for Clinical Medicine, Capital Medical University, Beijing 100069, China; Beijing Key Laboratory of Cancer Invasion and Metastasis Research, Beijing 100069, China; Key Laboratory of Epigenetic Regulation and Intervention, Institute of Biophysics, Chinese Academy of Sciences, Beijing 100101, China; University of Chinese Academy of Sciences, Beijing 100049, China; Chengdu ClonOrgan Biotechnology Co., Ltd., Chengdu 610041, China; School of Basic Medical Sciences, Capital Medical University, Beijing 100069, China; School of Basic Medical Sciences, Capital Medical University, Beijing 100069, China; Laboratory for Clinical Medicine, Capital Medical University, Beijing 100069, China; Beijing Key Laboratory of Cancer Invasion and Metastasis Research, Beijing 100069, China; School of Basic Medical Sciences, Capital Medical University, Beijing 100069, China; Laboratory for Clinical Medicine, Capital Medical University, Beijing 100069, China; Beijing Key Laboratory of Cancer Invasion and Metastasis Research, Beijing 100069, China; School of Basic Medical Sciences, Capital Medical University, Beijing 100069, China; Laboratory for Clinical Medicine, Capital Medical University, Beijing 100069, China; Beijing Key Laboratory of Cancer Invasion and Metastasis Research, Beijing 100069, China; School of Basic Medical Sciences, Capital Medical University, Beijing 100069, China; Laboratory for Clinical Medicine, Capital Medical University, Beijing 100069, China; Beijing Key Laboratory of Cancer Invasion and Metastasis Research, Beijing 100069, China; School of Basic Medical Sciences, Capital Medical University, Beijing 100069, China; Laboratory for Clinical Medicine, Capital Medical University, Beijing 100069, China; Beijing Key Laboratory of Cancer Invasion and Metastasis Research, Beijing 100069, China; School of Basic Medical Sciences, Capital Medical University, Beijing 100069, China; Laboratory for Clinical Medicine, Capital Medical University, Beijing 100069, China; Beijing Key Laboratory of Cancer Invasion and Metastasis Research, Beijing 100069, China; Chengdu ClonOrgan Biotechnology Co., Ltd., Chengdu 610041, China; Clinical Immunology Translational Medicine Key Laboratory of Sichuan Province, Sichuan Academy of Medical Sciences & Sichuan Provincial People’s Hospital, Chengdu 610000, China; School of Medicine, University of Electronic Science and Technology of China, Chengdu 611700, China; Key Laboratory of Epigenetic Regulation and Intervention, Institute of Biophysics, Chinese Academy of Sciences, Beijing 100101, China; University of Chinese Academy of Sciences, Beijing 100049, China; School of Basic Medical Sciences, Capital Medical University, Beijing 100069, China; School of Basic Medical Sciences, Capital Medical University, Beijing 100069, China; Laboratory for Clinical Medicine, Capital Medical University, Beijing 100069, China; Beijing Key Laboratory of Cancer Invasion and Metastasis Research, Beijing 100069, China

**Keywords:** CRISPR/Cas9, WGS, Genetic mutation, Xenotransplantation, Pig

## Abstract

Although xenotransplantation has been revolutionized by the development of genome-edited pigs, it is still unknown whether these pigs and their offspring remain genomically stable. Here, we showed that *GGTA1-*knockout (GTKO) pigs accumulated an average of 1205 genome-wide genetic mutations, and their filial 1 (F1) offspring contained an average of 18 *de novo* mutations compared with wild-type controls and their parents. The majority of mutations were in regions annotated as intergenic without altering protein functions, and none were located at predicted off-target sites. RNA sequencing analysis and phenotypic observations indicated that the accumulated mutations may have only a limited influence on GTKO pigs, and most of the mutations in the GTKO pigs could be attributed to the electrotransfection of plasmids into cells. This is the first report demonstrating that genetic mutations in genome-edited pigs are inherited stably by the next generation, providing a reference for the safe application and a standard approach to breed genome-edited pigs for xenotransplantation.

## Graphical abstract

**Figure:**
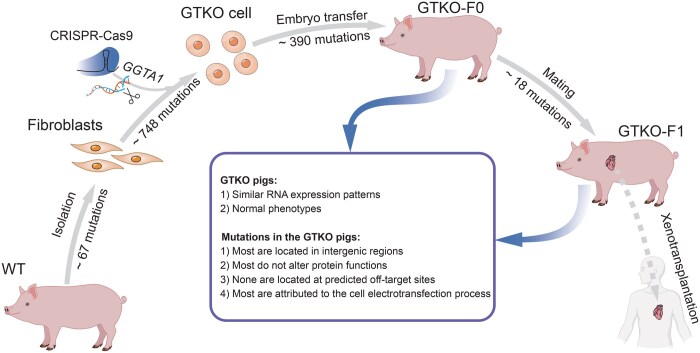

## Introduction

In recent years, the gap between the number of available organs and transplant demand has expanded. According to global statistics, more than 103,223 people are on the United States (US) transplant waiting list (https://www.organdonor.gov/; accessed August 12, 2025), and in China, approximately 378,500 candidates are on the transplant waiting list as of the end of 2023 [1]. In 2023, only 162,400 organ transplants were performed worldwide, fulfilling less than 8.3% of the global demand, while over 2 million patients awaited life-saving procedures (http://www.transplant-observatory.org; accessed July 15, 2025). In addition to the employment of traditional surgery and drugs, xenotransplantation, which aims to replace failing human organs with animal organs, has become the most viable method to address this issue [2].

During the latter part of the 20th century, the xenotransplantation community shifted its attention from primates to pigs as potential sources of organs and cells [3]. Pigs hold numerous advantages over nonhuman primates, such as faster reproduction, greater ease of breeding, lower costs, and anatomical characteristics and physiological indices more similar to those of humans [4]. Moreover, the use of pigs avoids the ethical issues caused by the use of nonhuman primate organs [5]. However, as a candidate xenograft source, pigs have two major drawbacks: cross-species transmission of porcine endogenous retroviruses (PERVs) [6,7] and immunological molecular incompatibilities [8]. Transmission of PERVs can be alleviated by knocking out viral genes that can be inserted into the human genome [9] or by directly using designed PERV-free pigs such as the Wuzhishan minipig [10]. When addressing immunologic barrier issues, administration of standard immunosuppressive medication has proven ineffective to prolong the survival of transplanted pig organs [11]. Editing (addition or elimination) of selected genes has been widely used to overcome immunologic obstacles [12,13]. A major step forward was taken when galactose-α1,3-galactose (α-1,3Gal) was identified as a compound responsible for evoking hyperacute rejection (HAR) of pig tissue by Old World primate recipients [14]. In 2002, researchers produced α-1,3-galactosyltransferase knockout (GTKO) pigs, which exhibited a significantly reduced rate of HAR in pig-to-primate organ transplantation [15]. Afterward, it was shown that xenografts from GTKO pigs notably prolonged the survival of baboons [16], and survival could be further improved by editing GTKO pig genomes to eliminate other genes involved in immune barriers [17]. More recently, pivotal advances have been achieved in pig-to-human preclinical studies, including successful pig kidney transplantation in living recipients [18], extended pig heart trials in human subjects [19], and the application of a pig liver xenograft in a human decedent [20]. Although xenotransplantation continues to face formidable immunological and coagulation barriers [1], these trials still show promise for the clinical application of gene-edited pig organs in xenografts.

The capacity to genetically modify pigs has undoubtedly promoted xenotransplantation. Nevertheless, new concerns about whether these gene modifications may induce alterations at the genomic or transcriptomic level remain largely unaddressed, leading to confusion and conflicts. For example, gene editing by CRISPR/Cas9 can induce large structural variants (SVs) and complex genomic rearrangements at the on-target site, as well as short mutations at the off-target sites [21]. In other cases, however, genome editing by CRISPR/Cas9 has been shown to be highly specific, with no undesired off-target or on-target mutations [22]. In addition, our previous studies also identified several microsatellite instabilities in the genome of CRISPR/Cas9-modified inbred mice [23]. In addition to off-target mutations caused by CRISPR/Cas9, pigs genome-edited for xenotransplantation may carry other mutational loads due to normal *in vivo* somatic mutations, because they are generated using cultured cells [24]. Moreover, genomic instability introduced during nuclear transfer reprogramming may induce mutations [25].

Referring to the 2017 draft guidance of the Food and Drug Administration (FDA) [26], developers of genome-edited animals should fully characterize the sites of the intended alterations and any additional changes (*e*.*g*., off-target alterations, unanticipated insertions, substitutions, or deletions), particularly within coding or regulatory regions. Unfortunately, most previous studies have primarily focused on the on-target and potential off-target sites predicted by computer programs, and the genome-wide identification of *de novo* mutations (DNMs) in pigs genetically edited for xenotransplantation, particularly throughout the processes of cell cloning and nuclear transfer, remains very limited. Here, we used GTKO pigs as founders to produce more xenotransplantation candidate animals and determined whether mutations in GTKO pigs could be inherited by their offspring during production, which is a valid but untested concern. Hence, it is necessary to characterize the genetic mutations that accumulate in genome-edited pigs and their offspring for xenotransplantation.

## Results

### GTKO pigs contain thousands of DNMs

**Figure 1:**
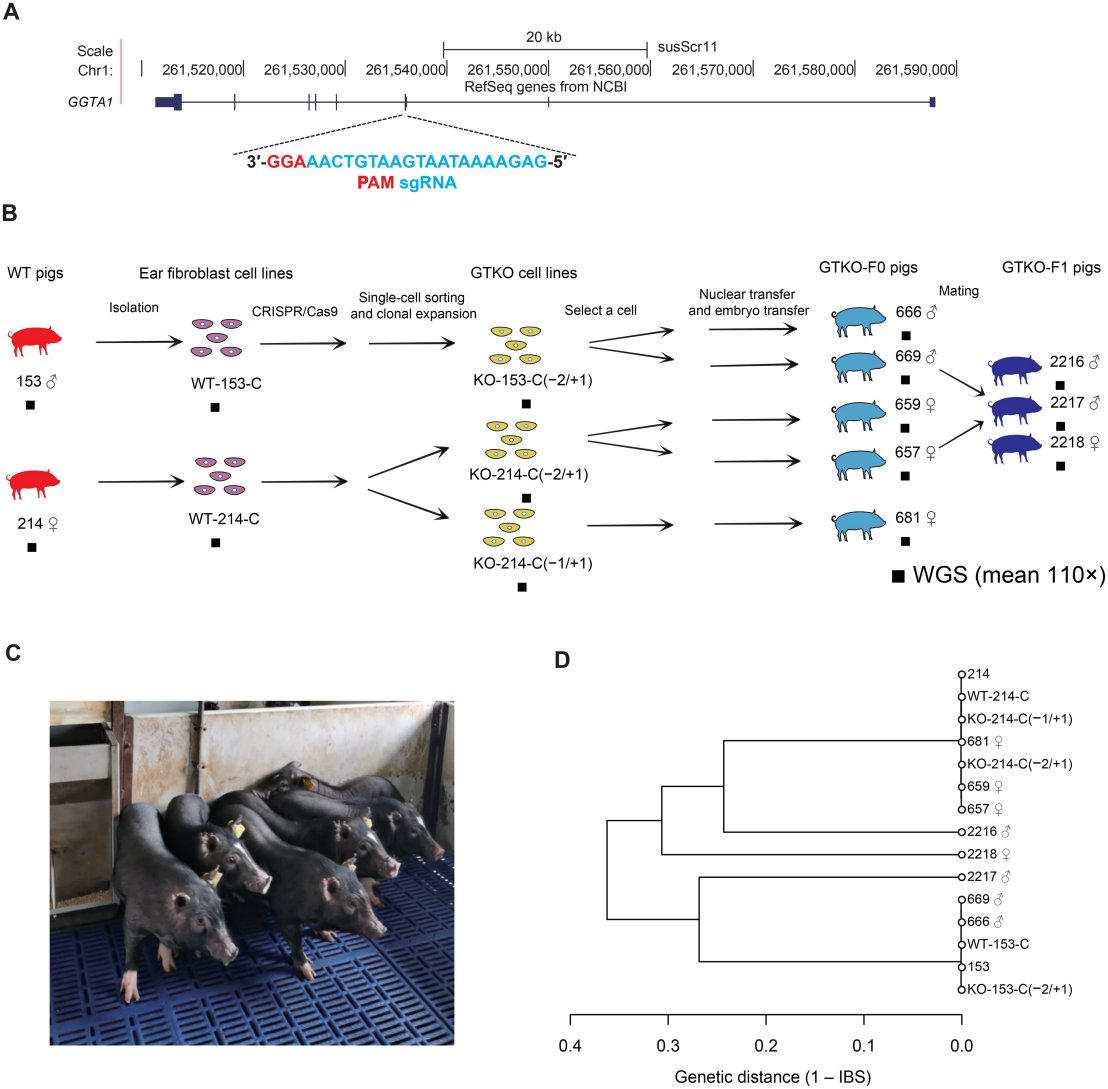
Experimental design **A**. Schematic overview of the design of the sgRNA targeting the *GGTA1* gene in Chinese Wuzhishan miniature pig. **B**. Workflow for generating GTKO-F0 and GTKO-F1 pigs. KO-153-C(−2/+1) denotes a CRISPR/Cas9-edited GTKO cell line from WT pig No. 153 (♂), carrying a 2-bp deletion and 1-bp insertion in *GGTA1*. KO-214-C(−2/+1) denote a CRISPR/Cas9-edited GTKO cell line from WT pig No. 214 (♀), carrying a 2-bp deletion (caused by a 4-bp deletion and a 2-bp insertion) and 1-bp insertion in *GGTA1*. KO-214-C(−1/+1) denotes a CRISPR/Cas9-edited GTKO cell line from WT pig No. 214 (♀), carrying a 1-bp deletion and 1-bp insertion in *GGTA1*. **C**. Photograph of GTKO piglets. **D**. Dendrogram showing the phylogenetic relationships (IBS distance) among the sequences analyzed in this study. Less similar sequences have branch points located closer to the root (left side) of the dendrogram. sgRNA, single-guide RNA; PAM, protospacer adjacent motif; WT, wild-type; GTKO, *GGTA1*-knockout; KO, knockout; WGS, whole-genome sequencing; IBS, identity-by-state.

We designed a systematic experimental framework (**Figure 1**A and B) to investigate the genetic mutations accumulated in GTKO pigs generated by CRISPR/Cas9-mediated genome editing for xenotransplantation. In short, GTKO-F0 pigs targeting exon 3 of the *GGTA1* gene were derived from two wild-type (WT) Chinese Wuzhishan minipigs (Figure 1C), and GTKO-F1 minipigs were produced by the mating of GTKO-F0 pigs that presented normal phenotypes (Figure S1; Table S1). We performed whole-genome sequencing (WGS) of five GTKO-F0 and three GTKO-F1 pigs and their cell lines at a mean depth of 110× to study how the genetic mutations arose. Genome sequences derived from the pigs with the same genotype were closely related and clustered together (Figure 1D). A detailed description of experimental design and sequencing data quality can be found in the Materials and methods section.

Since plasmid integration was reported in germline genome-edited cattle [27], we included the single-guide RNA (sgRNA)-Cas9 plasmid backbone sequence in our comparison of the WGS reads with the *Sus scrofa* 11.1 reference genome, and found an unexpected integration of the sgRNA-Cas9 plasmid into the genome (Chr1: 212,536,798) of GTKO-F0 pig No. 681 (Figure S2). We further confirmed this insertion by polymerase chain reaction (PCR)-based agarose gel analysis in all five GTKO-F0 pigs (Nos. 681–685) derived from the same *GGTA1*-edited cell line KO-214-C(−1/+1), indicating the integration of the origin [KO-214-C(−1/+1)] (Figure S3). Although no abnormal phenotype was observed in GTKO-F0 pig No. 681 after birth, implying that sgRNA-Cas9 plasmid integration did not affect the pig’s health, this integration was nonetheless considered an unexpected event, and care should be taken to avoid such integrations.

We identified DNMs that showed the gain of a new allele in each GTKO pig relative to its corresponding original WT genome, and the alleles were inheritable from parent to offspring. To characterize the genetic mutations accumulated in GTKO-F0 pigs, we employed the corresponding WT pigs as references. By combining three mutation callers (Mutect2 in GATK [28], Strelka2 [29], and VarScan2 [30]) and a series of reasonable filtering procedures (see Materials and methods), an average of 1205 DNMs [including single-nucleotide variants (SNVs) and insertions and deletions (indels)] per GTKO-F0 pig genome were identified (**Table 1**, Table S2). We also obtained the annotation information for each DNM, including intron_variant, 3_prime_UTR_variant, and missense_variant (Table S2), which were all fixed in heterozygous conditions. Mutations fixed in all GTKO-F0 pigs were located randomly in the genome. The percentages of SNV types were distributed randomly in each GTKO-F0 pig (Figure S4). GTKO pigs produced by the same route shared a comparable proportion of mutations. For example, GTKO-F0 pig No. 666 (814 DNMs) and No. 669 (1070 DNMs) shared 474 DNMs (58.2% and 44.3%, respectively). Similarly, GTKO-F0 pig No. 657 (1498 DNMs) and No. 659 (1490 DNMs) shared 1472 SNVs and indels (98.3% and 98.8%, respectively). The majority of total DNMs (5983 of 6022) were annotated as unlikely to change protein sequence by SnpEff software [31], using the corresponding Ensembl gene annotation file. On average, only eight DNMs per GTKO-F0 pig genome were predicted to potentially alter protein functions (Table 1). A total of 25 genes across all GTKO-F0 pigs were identified to carry mutations predicted to potentially alter protein functions. Among these, two (*EGFR* and *THBS1*) were associated with bladder cancer based on Kyoto Encyclopedia of Genes and Genomes (KEGG) pathway enrichment analysis [32] (Table 1). Four genes (*DCC*, *EGFR*, *LRIG3*, and *RUNX1*) mutated in GTKO-F0 pig No. 681 were previously found to be mutated in some human cancers [33] (Table 1). For example, *DCC* is a well-characterized human colorectal cancer suppressor gene, and *RUNX1* mutations are closely related to tumorigenesis in human leukemia [34] and breast cancer [35]. Although we did not observe any abnormal cancer-associated phenotype associated with the plasmid integration in GTKO-F0 pig No. 681 before it was eliminated at seven months of age (Table S3), it remains uncertain whether tumors or other abnormalities may develop in the future. In addition to cancer-related genes, GTKO-F0 pigs (Nos. 657 and 659) carried mutations in *RALGAPA1*, which has known roles in neurodevelopmental disorders with hypotonia [36]. We also investigated the presence of SVs (≥ 50 bp) with a combination of two SV calling methods (DELLY [37] and Manta [38]) and a series of reasonable filters (see Materials and methods). The results showed four identical high-confidence heterozygous tandem duplications in GTKO-F0 pig Nos. 666 and 669 (Table S4), which were predicted by SnpEff software [31] to cause structural changes in the corresponding genes [*HMG20A*, *MAN1C1*, *TUBD1*, *VMP1*, and ENSSSCG00000042071 (a predicted lncRNA gene)]. GTKO-F0 pig Nos. 666 and 669 behaved normally, with no phenotypes corresponding to the affected genes, possibly because the mutations are heterozygous. As microsatellite instability (MSI) is an important indicator of genomic instability [39], we calculated the MSIsensor score [40], which corresponds to the percentage of mutated microsatellite loci, to detect MSI status and compared it with that in WT pigs. The results showed that the genomes of GTKO-F0 pigs were all in a microsatellite-stable state (MSS; MSIsensor score < 3.5) (Table S5). In parallel, we performed read depth analysis across chromosomes and did not find any chromosomal aberrations in the genomes of GTKO pigs (Figure S5).

**Table 1:** DNMs occurring in GTKO-F0 pigs

GTKO pig ID	No. of DNMs (nonsynonymous/total)	Mutated gene
666	4/814	*DYRK1B*, *HNRNPUL1*, *UBE3B*
669	5/1070	*DYRK1B*, *HNRNPUL1*, *RYR3*, *TNRC18*
659	9/1490	*FBN3*, *MYLK*, *NUP188*, *OR3A1*,* PDE8B*, *PLOD2*, *PPF1A1*, *RALGAPA1*, *THBS1*
657	9/1498	*FBN3*, *MYLK*, *NUP188*, *OR3A1*,* PDE8B*, *PLOD2*, *PPF1A1*, *RALGAPA1*, *THBS1*
681	12/1150	*CDC14B*, *DCC*, *DIP2C*, *EGFR*, *EPHA6*, *ERC2*, LOC110259754, *LRIG3*, *LVRN*, *RUNX1*, *RYR3*, *SEC31B*
Average	8/1205	NA

*Note*: No. of nonsynonymous DNMs/No. of total DNMs in GTKO-F0 pigs against WT pigs were provided. Mutated genes refer to the genes with DNMs that were predicted to potentially alter protein functions in each individual. DNM, *de novo* mutation; GTKO, *GGTA1*-knockout; WT, wild-type; NA, not applicable.

To investigate DNMs in the offspring of GTKO-F0 pigs, we studied a family (pig Nos. 669, 657, 2216, 2217, and 2218). To our knowledge, the siblings produced as the GTKO-F1 generation are the first offspring of genome-edited pigs to be examined by WGS. We adopted the method described by Iyer et al. [41] to evaluate the statistical power of DNM detection. Given a mean sequencing depth of 110× and a minimum *de novo* allele frequency of 10% (see Materials and methods), the power to detect one DNM occurring in a single-cell zygote or two-cell-stage embryo was at least 99.98%. We performed genome-wide variant calling using four tools, (GATK [28], Platypus [42], Freebayes [43], and SAMtools [44]) with serial filtering, and then identified DNMs by TrioDeNovo software [45], referring to a previous DNM calling pipeline [22]. The combination of multiple calling tools is necessary to identify high-confidence variants, because the genome-wide variant calling performance of different tools varies. The variants called by all four tools were identified as high-confidence variants (Figures S6 and S7). Notably, the consistency of variant calling by the four different tools (> 68%) was similar to that reported in a study of Cas9-edited rhesus monkeys [18]. Finally, in addition to one high-confidence *de novo* indel, 17, 19, and 14 high-confidence *de novo* SNVs were identified in GTKO-F1 sibling pigs Nos. 2216, 2217, and 2218, respectively (**Table 2**). Only one mutation, in LOC100523897 in the GTKO-F1 pig No. 2217 genome, was predicted to alter protein function (Table 2, Table S6). All DNMs in the GTKO-F1 pigs were located randomly in the reference genome and did not overlap among offspring (Table S6). Taken together, these results show that the number and genome-wide distribution of DNMs among the three siblings are comparable. These DNMs in the offspring can be explained by the known spontaneous mutation rate in WT pigs (3.6 × 10^−9^ per site per generation; 95% confidence interval (CI) = 2.8 × 10^−9^–4.4 × 10^−9^), which corresponds to an expectation of 7–11 DNMs per generation [46]. We also evaluated whether there were *de novo* SVs (≥ 50 bp) based on two structural variant calling methods (DELLY [37] and Manta [38]) and multiple filters (see Materials and methods). No high-confidence *de novo* SVs were detected in the offspring (Table S7). Read depth analysis did not find any chromosomal aberrations in GTKO-F1 pigs (Figure S5). These results indicate that the genome of GTKO pigs is stably inherited across generations.

**Table 2:** DNMs present in GTKO-F1 pigs

GTKO-F0 pig ID (sire)	GTKO-F0 pig ID (dam)	GTKO-F1 pig ID (offspring)	No. of DNMs*	Mutated gene^#^
669	657	2216	0/18	–
		2217	1/20	LOC100523897
		2218	0/15	–

*Note*: * indicates No. of protein-function-altering DNMs/No. of total DNMs; ^#^ indicates mutated genes carrying DNMs that were predicted to potentially alter protein functions.

To examine the influence of these thousands of DNMs in GTKO pigs on molecular function, especially on gene expression, we performed RNA sequencing (RNA-seq) on blood samples from WT and GTKO pigs. Three GTKO-F0 pigs (Nos. 669, 657, and 659) and three GTKO-F1 pigs (Nos. 2216, 2217, and 2218) were included. Unfortunately, the corresponding WT pigs (Nos. 153 and 214) were not sampled due to management constraints. Therefore, four WT pigs (Nos. 872, 876, 937, and 950) which were genetically close to Nos. 153 and 214 (same species and breed in the same facility) were included; the grandfather of these pigs was WT pig No. 153. All pigs were fed in the same environment. In total, we obtained an average of 60 million raw reads for each sample, with Q30 RNA paired-end reads higher than 90% (Table S8). We detected the expression of three PERV subtypes in the blood samples of these pigs, and found the expression of PERV-A and PERV-B subtypes but not PERV-C (Figure S8), confirming that these pigs were PERV-C free. This mitigates the special risk of the transmission of PERVs via xenotransplantation [47]. Then, we observed that the relative expression levels of most genes were very similar, with an average Pearson’s correlation coefficient of expression levels among the samples > 0.998 (Figures S9 and S10A). It is worth noting that normal expression levels of the *GGTA1* gene were maintained in GTKO pigs relative to WT pigs (Figure S10B and C), indicating that GTKO pigs share similar gene expression patterns in the blood with WT pigs. With |log_2_ fold change| > 1 and an adjusted *P* value < 0.01 as cutoffs, we observed 12 upregulated genes and 7 downregulated genes in GTKO-F0 pigs compared with WT pigs (Figure S10B), as well as 152 upregulated genes and 63 downregulated genes in GTKO-F1 pigs compared with WT pigs (Figure S10C). Gene Ontology (GO) and KEGG pathway enrichment analyses showed that the significantly differentially expressed genes (DEGs) of GTKO-F0 pigs were not enriched in any specific pathways. The DEGs of GTKO-F1 pigs were enriched in pathways such as metabolic pathways, which may exert a minor influence on pig health and xenotransplantation (Figure S10D). Taken together, these results show that accumulated mutations may have a limited impact on the healthy phenotypes of GTKO pigs.

### DNMs in GTKO pigs arise through multiple mechanisms

GTKO-F0 pigs were initially developed from a single cell. Thus, mutations may originate in fibroblast lines or GTKO cell lines at a low frequency. We analyzed the genome sequences of the corresponding fibroblast lines and GTKO cell lines to investigate how these thousands of DNMs accumulated during the generation of GTKO-F0 pigs (see Materials and methods). Given the mean WGS depth of 110× (Table S9), DNMs with frequency > 1% preexisted in fibroblast lines or GTKO cell lines. Of all 1205 observed DNMs fixed in GTKO-F0 pig genomes on average, 67 existed and were fixed in fibroblast lines, and 748 existed and were fixed in GTKO cell lines on average (**Table 3**). Surprisingly, on average, 358 DNMs per GTKO-F0 pig genome could not be observed in either the corresponding fibroblast lines or the corresponding GTKO cell lines. Considering the reports of genomic instability during reprogramming by nuclear transfer [25,48,49], we speculated that these unassigned DNMs occurred during or shortly after nuclear transfer and were then fixed in the GTKO-F0 pigs. In addition, among the four heterozygous tandem duplications observed in the GTKO-F0 pig Nos. 666 and 669, three were originally presented in GTKO cell lines, and one occurred during or shortly after nuclear transfer (Table S4). The genomes of the cell lines were all in the MSS state (Table S5), and no chromosome-level changes were observed in either fibroblast lines or GTKO cell lines (Figure S5).

**Table 3:** DNMs initially arose in fibroblast and GTKO cell lines

GTKO pig ID	Existed firstly in fibroblast cell lines		Existed firstly in GTKO cell lines		Existed firstly during or after nuclear transfer	
	No. of DNMs*	Mutated gene^#^	No. of DNMs*	Mutated gene^#^	No. of DNMs*	Mutated gene^#^
666	0/60	−	4/509	*DYRK1B*,* HNRNPUL1*,* UBE3B*	0/245	−
669	0/60	−	4/579	*DYRK1B*,* HNRNPUL1*,* RYR3*	1/431	*TNRC18*
659	0/76	−	4/958	*FBN3*,* MYLK*,* THBS1*,* OR3A1*	5/456	*PDE8B*,* NUP188*,* PLOD2, PPFIA1, RALGAPA1*
657	0/76	−	4/963	*FBN3*,* MYLK*,* THBS1*,* OR3A1*	5/459	*PDE8B, NUP188, PLOD2, PPFIA1, RALGAPA1*
681	1/61	*LRIG3*	4/731	*EPHA6*,* SEC31B*,* CDC14B*, LOC110259754	7/358	*DCC*,* DIP2C*,* EGFR*,* ERC2*, *LVRN*,* RUNX1*,* RYR3*
Average	0/67	−	4/748	−	4/390	−

*Note*: * indicates No. of protein-function-altering DNMs/No. of total DNMs; ^#^ indicates mutated genes carrying DNMs that were predicted to potentially alter protein functions.

**Figure 2:**
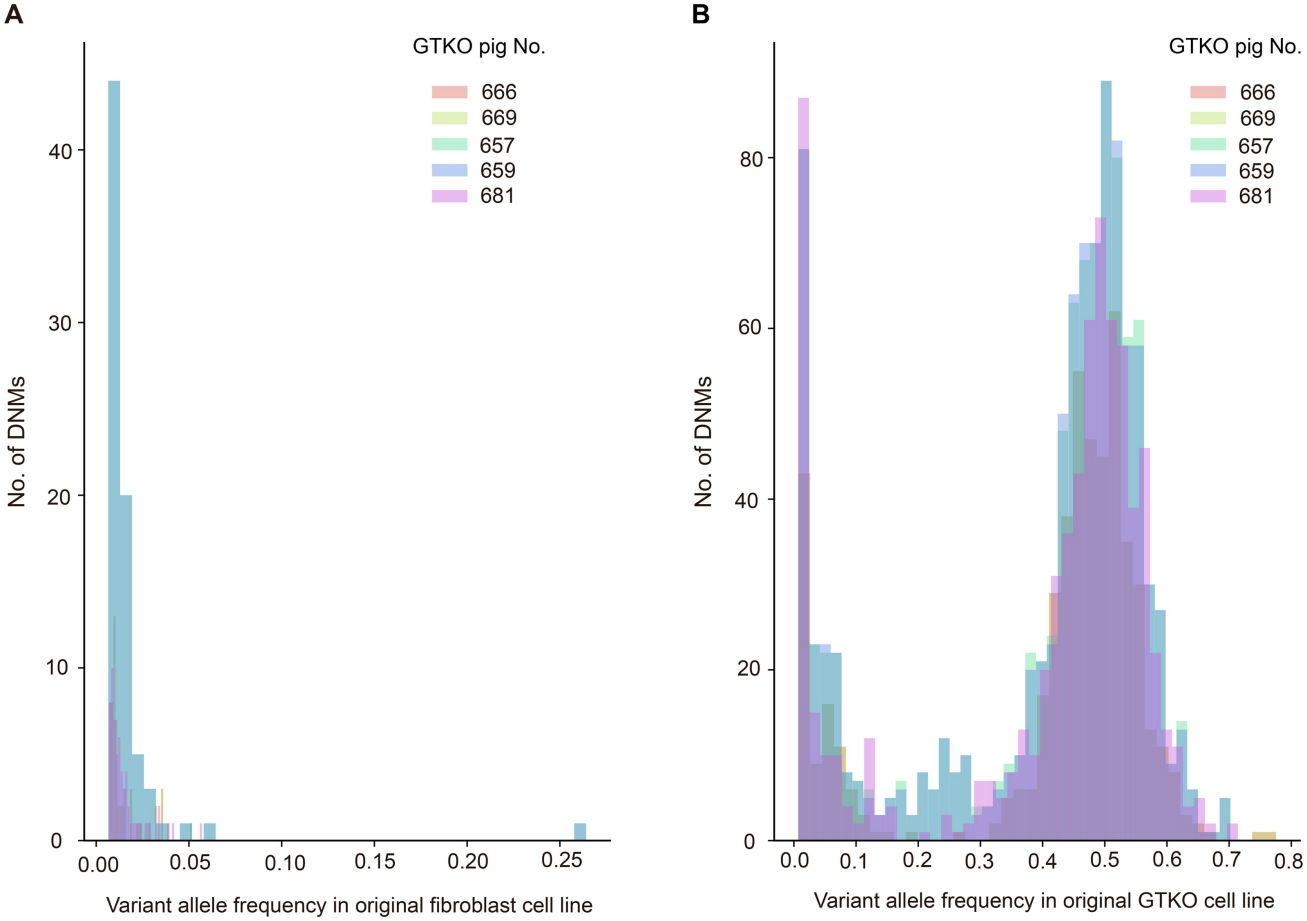
Variant allele frequency of DNMs in the original fibroblast and GTKO cell lines **A**. Mutations initially arose in the original fibroblast cell line. **B**. Mutations initially arose in the GTKO cell line. DNM, *de novo* mutation.

Mutations that initially appeared in distinct wild-type fibroblast lines and were ultimately fixed in the GTKO-F0 pig genomes were not shared across the original cell lines. For example, mutations preexisting in the WT-153-C and WT-214-C fibroblast lines were not shared. However, mutations existing first in the same fibroblast line and finally fixed in the GTKO-F0 pig genomes were shared. For example, 60 DNMs that existed first in the WT-153-C fibroblast line and were fixed in the GTKO-F0 pig Nos. 666 and 669 were identical. Mutations that existed first in WT fibroblast lines all occurred at a low frequency (**Figure 2**A), indicating that these mutations were normal somatic mutations that occurred during *in vitro* fibroblast culture.

Mutations that existed in the same GTKO cell line and were fixed in the GTKO-F0 pig genomes were largely shared. For example, mutations that existed in the KO-153-C(−2/+1) cell line and were fixed in the GTKO-F0 pig Nos. 666 and 669 were 81.3% and 71.5% shared, respectively. The number of mutations that existed first in GTKO cell lines was significantly greater than that in fibroblast lines (normal culture mutational load) (*P* < 0.05; Mann–Whitney test). A proportion of mutations in the GTKO cell lines were present at a low frequency (Figure 2B), indicating that these mutations were introduced at the later stages of cell line culturing. Other mutations in GTKO cell lines existed at nearly 50% frequency, indicating that these mutations were present in almost all cells and were introduced in progenitor cells. Therefore, some of the detected mutations did not arise during *in vitro* GTKO cell culture. This result raises the possibility that a significant number of mutations occur during or shortly after genome editing and then become fixed during colony picking and expansion.

To investigate whether DNMs existing in the GTKO cell lines were off-target mutations, we measured the targeting specificity of the CRISPR/Cas9 technology. We first examined the on-target mutation of *GGTA1* in GTKO-F0 pigs (Nos. 657, 659, 666, 669, and 681) and found that the site was successfully modified by the designed CRISPR/Cas9 system in all cases. We also found that these on-target mutations indeed existed in the corresponding GTKO cell lines [KO-153-C(−2/+1), KO-214-C(−2/+1), and KO-214-C(−1/+1)], indicating that on-target mutations were passed to GTKO-F0 pigs produced by somatic cell nuclear transfer (SCNT) (Figure S11A). The inheritance of on-target mutations from parental GTKO-F0 pigs (Nos. 669 and 657) to the GTKO-F1 offspring (Nos. 2216, 2217, and 2218) was also in line with Mendelian inheritance (Figure S11A), indicating that on-target mutations were stably passed across generations. We further observed consistent results at the RNA level (Figure S11B). In addition, the observed on-target variant allele fractions (VAFs) both at the cell level and at the individual GTKO pig level were nearly 0.5, indicating that the on-target mutations are not mosaic. No other genome sequence changes, including large SVs at the intended on-target site, were observed in any of the genomes. To check whether mutations existing in GTKO cell lines resulted from CRISPR/Cas9, we performed off-target analysis. Cleavage by the widely studied *Streptococcus pyogenes* Cas9 ortholog (SpCas9) is mediated by a 20-nt segment of a sgRNA complementary to a target DNA sequence that precedes a protospacer adjacent motif (PAM), where Cas9 is recruited [50]. With the employment of Cas-OFFinder software [51], which allows up to 3 mismatches with 1-bp indel and up to 4 mismatches with no indels between the on-target and off-target sites, we predicted 11,634 potential off-target locations for the *GGTA1* sgRNA across the reference genome, which were randomly distributed (Table S10; Figure S12). However, no mutation that accumulated in GTKO cell lines or GTKO pigs (SNVs, indels, and SVs) was found in any predicted off-target site (**Table 4**). Hence, no off-target effects were detected for the designed sgRNA for GTKO pigs at the genome-wide level. That is, mutations preexisting in the same GTKO cell line did not arise as side effects of gene editing.

**Table 4:** Summary of mutations in the off-target regions predicted by Cas-Offinder

Sequence		No. of nucleotide mismatches					No. of predicted off-target sites
		0	1	2	3	4	
CRISPR sites	Target only	1*	0	8	99	1227	1335
	Target +1 (DNA bulge)	1	13	152	2343	–	2509
	Target −1 (RNA bulge)	2	20	518	7250	–	7790
	Overall	4	33	678	9692	1227	11,634
Genome mutations	GTKO cell lines	KO-153-C(−2/+1)	1*	0	0	0	0
		KO-214-C(−1/+1)	1*	0	0	0	0
		KO-214-C(−2/+1)	1*	0	0	0	0
	GTKO-F0 pigs	669	1*	0	0	0	0
		666	1*	0	0	0	0
		659	1*	0	0	0	0
		657	1*	0	0	0	0
		681	1*	0	0	0	0
	GTKO-F1 pigs	2216	1*	0	0	0	0
		2217	1*	0	0	0	0
		2218	1*	0	0	0	0

*Note*: * indicates the on-target sgRNA. sgRNA, single-guide RNA.

**Figure 3:**
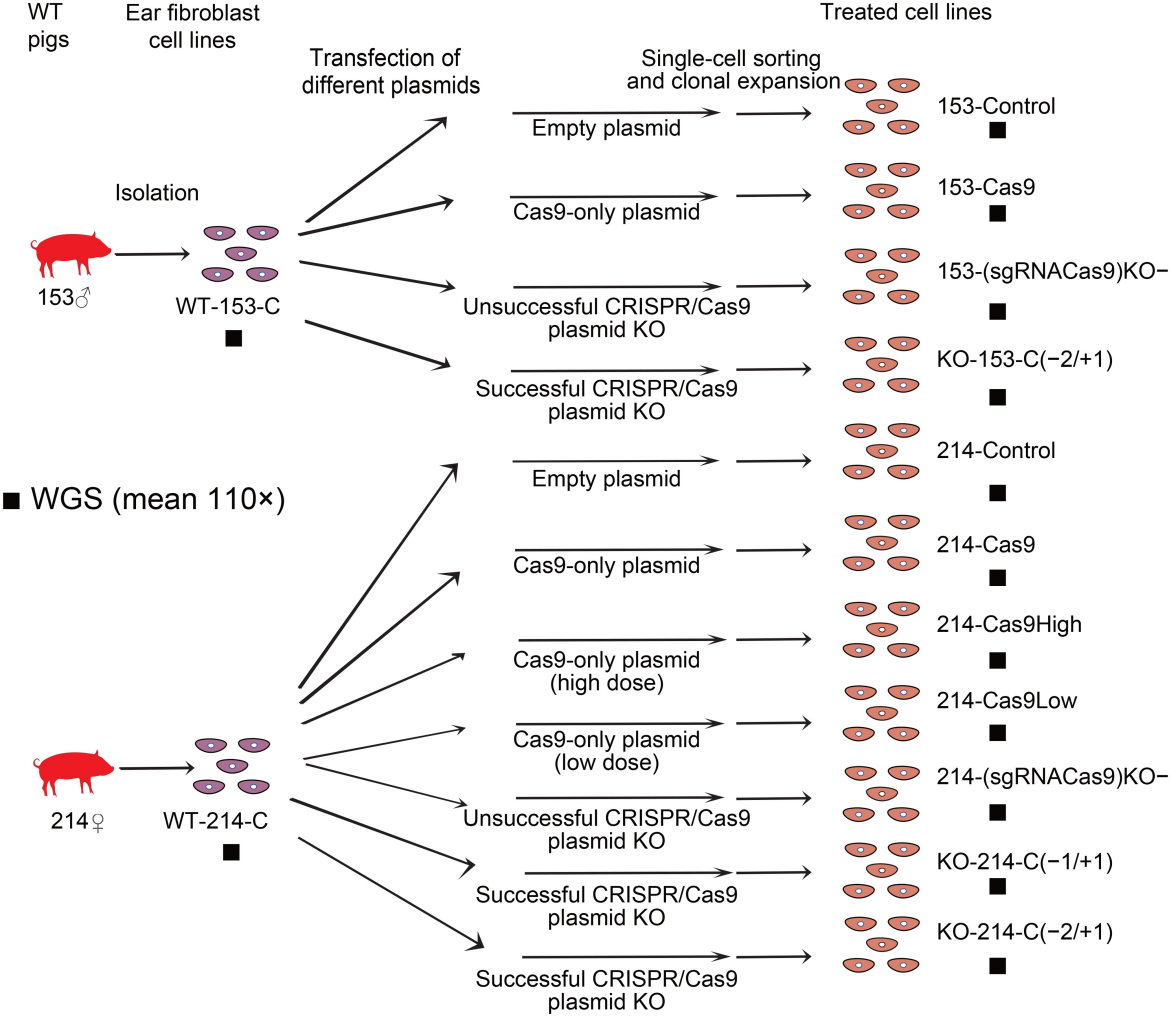
Schematic overview of different CRISPR/Cas9 systems WT ear fibroblasts (WT-153-C/WT-214-C) were transfected with: empty plasmid (153-Control/214-Control); Cas9-only plasmid (153-Cas9/214-Cas9); Cas9-only plasmid (high dose) (214-Cas9High); Cas9-only plasmid (low dose) (214-Cas9Low); unsuccessful CRISPR/Cas9 plasmid KO [153-(sgRNACas9)KO−/214-(sgRNACas9)KO−]; and successful CRISPR/Cas9 plasmid KO [KO-153-C(−2/+1)/KO-214-C(−1/+1)/KO-214-C(−2/+1)]. All lines underwent WGS (110× depth) to assess Cas9/sgRNA dosage effects on genomic integrity. Indel notation (*e*.*g*., −2/+1) indicates mutation patterns.

We extended this analysis to assess whether the CRISPR/Cas9 plasmid was mutagenic and responsible for the hundreds of DNMs that existed in the GTKO cell lines. We designed experiments to explore specific potential factors that could influence genome stability, including the dose and presence of Cas9 and sgRNA. Referring to the production of the GTKO-F0 pigs, we established the treated cell lines: “empty plasmid” (153-Control and 214-Control), “Cas9-only plasmid” (153-Cas9 and 214-Cas9), “Cas9-only plasmid (high dose)” (214-Cas9High), “Cas9-only plasmid (low dose)” (214-Cas9Low), and “unsuccessful CRISPR/Cas9 plasmid KO” [153-(sgRNACas9)KO− and 214-(sgRNACas9)KO−], and performed WGS with a mean depth of 110× on the DNBSEQ-T7 platform (**Figure 3**; Table S9). A “successful CRISPR/Cas9 plasmid KO” group [KO-153-C(−2/+1), KO-214-C(−2/+1), and KO-214-C(−1/+1)] was also included as the positive control. The WGS data exhibited high read quality with Q30 > 90%, duplicate percentage < 6%, and properly paired reads > 98% (Table S9). We first confirmed that the *GGTA1* gene was not edited by the Cas9 protein in any treated groups, especially the “unsuccessful CRISPR/Cas9 plasmid KO” group. Across all treated cell lines, we identified sites that showed the gain of a new allele relative to their corresponding matched progenitor fibroblast genome. Notably, hundreds of high-confidence DNMs were detected in the “empty plasmid” group, the “Cas9-only plasmid” group, the “Cas9-only plasmid (high dose)” group, the “Cas9-only plasmid (low dose)” group, and the “unsuccessful CRISPR/Cas9 plasmid KO” group (**Table 5**, Table S11), which were all randomly distributed in different chromosomes. Further analysis showed that there was no overlap of these mutations among groups. Only a few mutations per treated cell line genome were predicted to alter protein function (Table 5). No high-confidence SV or chromosomal aberration was observed in any treated groups (Table S4). The number, distribution, and predicted effects of mutations in all groups were comparable, indicating that these mutations are not associated with different components of the CRISPR/Cas9 plasmid.

**Table 5:** DNMs observed in treated cell lines

Group	Treated cell line	No. of DNMs*	Mutated gene^#^
Empty plasmid	153-Control	3/471	*TCP11L2*, *KIF21A*, *GNB1*
	214-Control	8/779	*TKTL2*, *APP*, *PHF24*, *ZNF555*, *NEB*, *METAP1*, *HTR1F, ZFAND4*
Cas9-only plasmid	153-Cas9	8/589	*CTF1*, *FAM183A*, *PIEZO2*, *ZNF395*, *EXD2*, *ZNF444*, ENSSSCG00000042643, *KAT2B*
	214-Cas9	5/816	*FREM1*, *TMC1*, ENSSSCG00000050360, *WDR37*, *CRIM1*
Cas9-only plasmid (low dose)	214-Cas9Low	1/491	*ANKRD31*
Cas9-only plasmid (high dose)	214-Cas9High	5/616	*PHF21B*, *POLI*, *ATAD2B*, *CCDC14*, *C8A*
Unsuccessful CRISPR/Cas9 plasmid KO	153-(sgRNACas9)KO−	10/847	*ZNF395*, ENSSSCG00000051581, *SYNGAP1*, ENSSSCG00000010426, *PRDM7*, *ZNF395*, ENSSSCG00000038947, *TRAV3*, *KIF19*, *FGFR3*
	214-(sgRNACas9)KO−	8/645	ENSSSCG00000008892, ENSSSCG00000041725, *CAPN5*, ENSSSCG00000016226, *LPCAT1*, *ZNF704*, *SLC12A1*, *CACNA1G*
Successful CRISPR/Cas9 plasmid KO	KO-153-C(−2/+1)	2/518	*HNRNPUL1*
	KO-214-C(−2/+1)	5/920	*LRRIQ3*, *FBN3*, *OR3A1*, *MYLK*, *THBS1,*
	KO-214-C(−1/+1)	5/685	*EPHA6*, *CDC14B*, LOC110259754, *SEC31B*, *LHX5*

*Note*: * indicates No. of protein-function-altering DNMs/No. of total DNMs; ^#^ indicates mutated genes carrying DNMs that were predicted to potentially alter protein functions.

**Figure 4:**
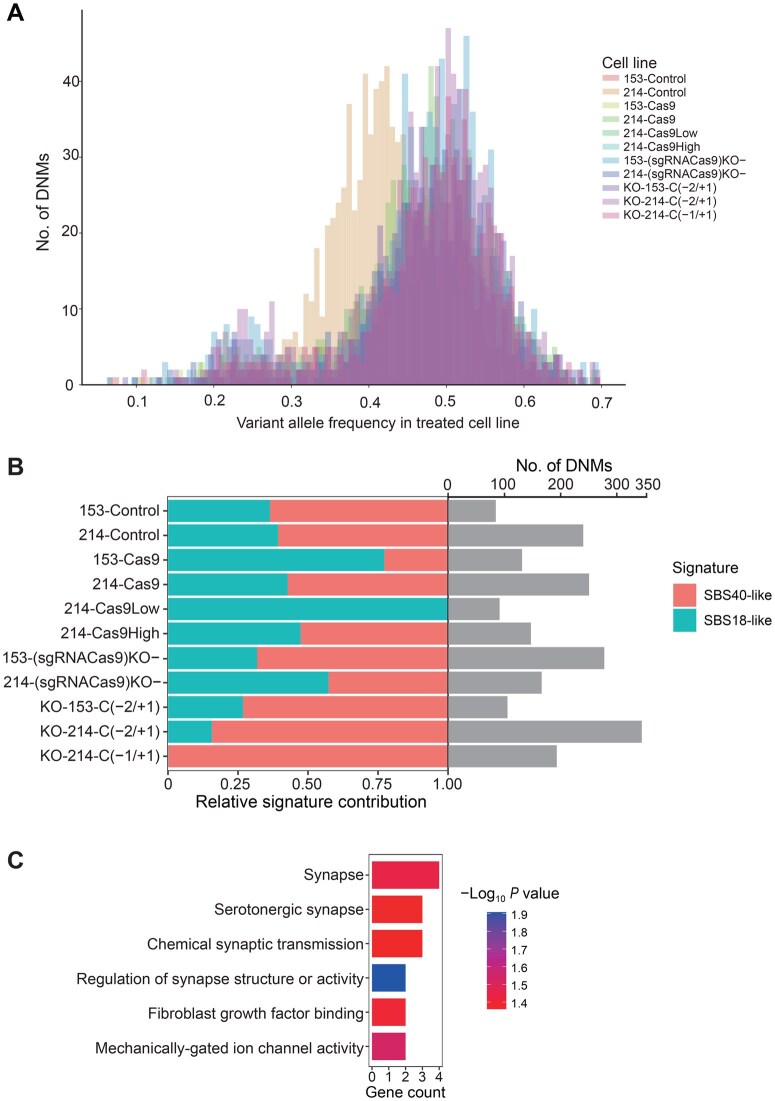
Characterization of DNMs occurring across treated cell lines **A**. Variant allele frequency of DNMs in treated cell lines. **B**. Relative enrichment of SNV mutational signature activities in treated cell lines. The number of DNMs assigned to signatures is shown on the right. **C**. GO and KEGG enrichment analyses of mutated genes across all treated cell lines. SNV, single-nucleotide variant.

A large proportion of mutations were heterozygous at approximately 50% frequency, indicating that these mutations should be present in almost all cells in the treated cell lines and were likely introduced in the progenitor cell (**Figure 4**A). Some DNMs preexisted at a low frequency (< 0.2), indicating that these mutations were introduced at later stages of treated cell line development (Figure 4A). These results also raise the possibility that some mutations occurred during or shortly after genome editing and then became fixed during colony picking and expansion, while others occurred during subsequent culturing in all treated cell lines.

To infer the processes underlying the mutational patterns of somatic alterations in the genome, we performed mutation signature analysis of DNMs that occurred in all treated cell lines. Mutation signature analysis revealed the existence of an SBS18-like pattern and an SBS40-like pattern (Figure 4B). The SBS18-like pattern was similar to the SBS18 mutational signature reported in the Catalog of Somatic Mutations in Cancer (COSMIC) database [32], with a cosine similarity of 0.88. The SBS40-like pattern was similar to the SBS40 mutational signature reported in the COSMIC database, with a cosine similarity of 0.82. The number of mutations attributed to SBS40 is correlated with patient age for certain types of human cancers, suggesting that they may be spontaneous somatic mutations. The proposed etiology of the SBS18 signature in the COSMIC database is damage caused by reactive oxygen species (ROS) [32]. Previous studies have reported that electrotransfection can lead to cell cycle arrest and apoptosis and may cause damage to genomic DNA, presumably due to ROS generation during pulse application [52]. The majority of mutations induced by ROS appear to involve modification of guanine, leading to C > A/G > T transversions [53]. Considering that the most common SNV type across all groups was C > A/G > T (Figure S4) as well as the attribution of the SBS18 signature, we infer that the mutations may have been induced by the physical stimulation for plasmid introduction rather than by gene editing *per se*. This also suggests that optimizing the delivery method of the Cas9 protein could help prevent the occurrence of hundreds of mutations during the production of GTKO pigs for xenotransplantation.

We further investigated whether these mutated genes could confer a common functional advantage through GO and KEGG pathway enrichment analyses. We found that some mutated genes were enriched in synapse-related GO terms and pathways, such as “regulation of synapse structure or activity (GO:0050803)” (Figure 4C). In light of the reports that ROS accumulation can be harmful to synaptic plasticity [54], we hypothesize that the electrotransfection of cells is responsible for the occurrence of those mutations in all treated cell lines.

## Discussion

In this study, we systematically investigated the DNMs that accumulated in five genome-edited pigs (GTKO-F0 pigs) generated for xenotransplantation and their three offspring, using the original WT pigs as a baseline. Using high-depth WGS, we explored where and how these genetic mutations accumulated. Although thousands of genetic mutations were identified in the GTKO-F0 pigs and their subsequent generations, our transcriptomic and phenotypic analyses revealed that these mutations did not affect gene expression or cause obvious phenotypic abnormalities. Furthermore, we demonstrated that the DNMs in the GTKO-F0 pigs arose in fibroblast lines and GTKO cell lines during and after nuclear transfer.

The safety of gene-edited pigs and their progeny, specifically the genome stability of founder animals of gene-edited pig populations used as xenotransplantation candidates, is a key concern. Ensuring safety is one of the primary goals of this industry and is therefore significant. Thus, for the first time, we explored the inheritance of CRISPR/Cas9-edited genomes across generations in pigs. Our WGS analysis revealed that the on-target mutations and DNMs that occurred in the GTKO-F0 pigs were stably inherited by the GTKO-F1 generation. This important finding advances our understanding of how the mutations in CRISPR/Cas9-edited GTKO-F0 pigs are inherited by individuals in the next generation, which has very recently been investigated by WGS, albeit at a lower average depth, in CRISPR/Cas9-edited zebrafish [55], sheep [56], and goats [57] (developed from fertilized eggs microinjected with CRISPR/Cas9 system components), as well as in a hornless bull that was genome-edited by TALEN [58]. In addition, RNA-seq data showed that the gene expression profiles of GTKO-F1 offspring were similar to those of GTKO-F0 and WT pigs. All offspring were healthy and phenotypically normal. Our meticulous confirmation of stable inheritance from GTKO pigs supports the value of gene-edited pigs, providing the first scientific evidence that CRISPR/Cas9-edited pigs could be expanded for xenotransplantation through traditional breeding methods, which are considerably less costly than cloning.

Although the CRISPR/Cas9 system is poised to become an era-marking gene editing tool broadly applied in various fields, and even in clinical contexts [59], biological safety concerns remain daunting. Here, we report that CRISPR/Cas9-edited GTKO pigs harbor a few accumulated mutations, exhibit similar RNA expression patterns, and show normal phenotypes, compared with WT pigs. The number of genomic mutations found in GTKO-F0 pigs is comparable to that reported in a previous study [49], albeit at varying levels. To disentangle the causal drivers of these DNMs in GTKO-F0 pigs, we comprehensively characterized the mutations from different aspects. Our data indicate that primitive fibroblasts contribute little to the instability of the genome, whereas hundreds of mutations may arise during the process of developing GTKO cell lines and GTKO pigs, implicating CRISPR/Cas9 and SCNT technology, respectively. A more detailed analysis of the influence of the CRISPR/Cas9 system on the genome stability of the cells derived from monoclonal screening reflects that the introduction of any plasmid, whether containing CRISPR/Cas9 components or not, elicits a similar number of variations. According to previous reports, these mutations are mostly considered low risk, with SNVs predominantly C > A/G > T, potentially related to the ROS produced during electrotransfection [52,60]. Similar mutational profiles were also found in cells transduced with different plasmids, further corroborating conclusions drawn from genomic analysis. Generally, mutations identified in GTKO-F0 pigs were evaluated as low risk based on their features, including type, location, and predicted effect on gene function. Furthermore, our RNA-seq data suggest that the genomic changes do not affect gene expression. Although the CRISPR/Cas9 nuclease system is reported to induce chromosomal deletions (megabase-scale) in human embryos [61], cell lines [62], and zebrafish [63], neither chromosomal aberrations or deletions in any cell line or animal nor any predicted off-target effects (another major concern related to CRISPR/Cas9 [64]), were observed in our experiments, indicating that our sgRNA design achieved high targeting specificity. Collectively, these results suggest that CRISPR/Cas9 and SCNT technologies do not introduce additional genomic risk, partially indicating the biological safety and potential efficacy of GTKO pigs for xenotransplantation. Given MSI high (MSI-H) is a hallmark of the genome with hypermutational phenotypes [65], we focused on MSI in genetically modified mice for decades, and found genomic instability events such as MSI in a dozen CRISPR/Cas9-edited mice at both selected off-target microsatellite loci [23] and loci linked to targeted genes [65] at significantly higher levels than observed in mice that were mutated using traditional gene-targeting approaches. However, we did not detect MSI events in the GTKO pigs, according to the MSIsensor scores from the WGS data. All GTKO samples, including cell lines and animal samples, were in MSS at the genome level, reflecting overall genomic stability.

**Figure 5:**
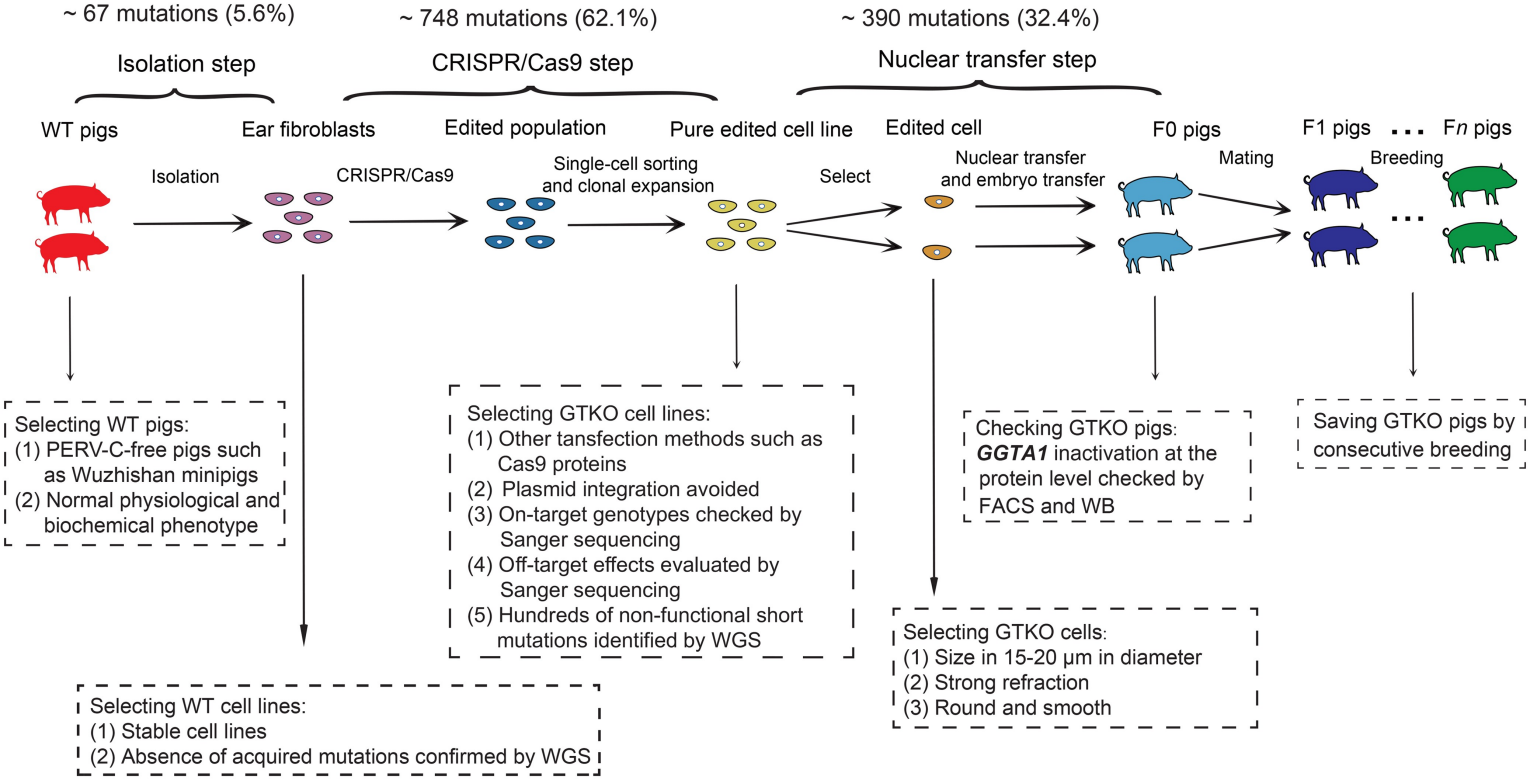
Proposed workflow for generating GTKO pigs for xenotransplantation FACS, fluorescence-activated cell sorting; WB, Western blotting.

The success of gene editing in pigs partially decreases the potential risks of both viral transmission and immunological incompatibility in xenotransplantation and brings hope for alleviating human organ shortages. However, achieving safe and effective gene editing on a large scale remains a challenge. Our evaluation, based on genomic, transcriptomic, and phenotypic data, provides powerful evidence for the safety of GTKO pigs for xenotransplantation compared with WT pigs. We examined each key step in the production of GTKO pigs and identified several opportunities to improve both the safety and success of the production of GTKO pigs (**Figure 5**). First, we recommend that donor pigs for xenotransplantation should be PERV-C-free pigs, such as Chinese Wuzhishan pigs, which is consistent with the recommendation of the International Xenotransplantation Association [66]. Next, we suggest that cell lines should be isolated from the tissues of donor pigs with stable normal physiological and biochemical phenotypes, and it should be confirmed that no mutations are introduced at the isolation step (Figure 5). Our third and most crucial suggestion is to optimize transfection methods to reduce genome instability during CRISPR/Cas9 plasmid introduction; alternatively, monitoring of this step should be strengthened efficiently. In this process, it is necessary to carefully select edited cells by assessing on-target effects, avoiding plasmid integration, and evaluating off-target effects using PCR and Sanger sequencing. Although the CRISPR/Cas9 system (*i.e.*, mRNA or protein) is delivered to zygotes at the single-cell stage [67], mosaicism is a common problem in animal genome editing by microinjection. Here, we employed SCNT technology, a method used to produce cloned animals that could ideally avoid the occurrence of mosaicism. Finally, we suggest rechecking on-target effects at the protein level after producing GTKO pigs and their siblings. If all of these steps are successfully implemented, GTKO pigs can be considered genetically stable. In addition, considering the estimated 34–42 doublings between the fibroblast line and the GTKO cell line and the previously published typical mutation rate [68], approximately 52–64 mutations are expected to occur naturally during this process, which aligns with our observations. Taken together, these results show that the biosafety of the GTKO pigs compared with that of WT pigs should be confirmed reliably by careful quality control of the production process.

Nevertheless, there are some aspects that need to be further investigated. First, we used a single gene-edited pig as a model xenotransplant pig. Multigene editing in pigs by CRISPR/Cas9 is more promising for clinical application, but whether the influence of multigene editing is similar to that of single-gene editing has not yet been determined. Second, while our short-read sequencing enabled robust SNV/indel detection, it could not identify the mutations in short tandem repeats (which harbor mutation frequency as high as point mutations). Thus, future studies employing long-read sequencing would provide a more comprehensive assessment of SVs in CRISPR-edited genomes. Furthermore, although the Wuzhishan pig species is popular in xenotransplant research, additional comparative investigations with various breeds, such as Bama, Gottingen, and Yorkshire pigs, that have been used in practical xenotransplant research, are needed. Finally, while we analyzed the transcriptomes of edited pigs in this study, proteomic analyses and functional verification are necessary in the future.

## Materials and methods

### Experimental design and sequencing data quality

To systematically investigate the genetic mutations that accumulate in genome-edited pigs for xenotransplantation, we used the Chinese Wuzhishan GTKO minipig as a representative model; these model pigs are naturally PERV-C free to prevent PERV transmission to the human recipients [10]. To create Chinese Wuzhishan GTKO minipigs for xenotransplantation, we designed an sgRNA to target exon 3 of the *GGTA1* gene (Figure 1A), which encodes galactose-α1,3-galactose [69]. We electroporated CRISPR/Cas9 reagents with the designed sgRNA from *S. pyogenes* into fibroblasts isolated from the ear tissue of two WT Chinese Wuzhishan minipigs (Nos. 153 and 214). Next, genetic modification of *GGTA1* and off-target effects were evaluated using PCR and Sanger sequencing (Table S12). Then, we used clones validated to have high KO efficiency (100% and 95% in fibroblasts of WT pig Nos. 153 and 214, respectively; Table S13) to successfully generate GTKO-F0 pigs by SCNT with an average SCNT success rate of 2.04% (Table S14). We mated GTKO-F0 pigs to produce GTKO-F1 offspring (Figure 1B and C). GTKO-F1 pigs were born uneventfully and healthy. We compared the health of 15 GTKO-F1 pigs with that of WT pigs in terms of body weight (Figure S1A), blood index (Table S1), and litter performance (Figure S1B) at approximately six months of age. No differences (*P* > 0.05) were observed between GTKO-F1 and WT pigs.

To investigate the genetic mutations that accumulated in GTKO pigs compared with the original WT pigs, we studied five GTKO-F0 pigs: pig Nos. 666 (♂) and 669 (♂) were derived from cell line KO-153-C(−2/+1); pig Nos. 657 (♀) and 659 (♀) were derived from KO-214-C(−2/+1), and pig No. 681 (♀) was derived from KO-214-C(−1/+1) (Figure S1B; Table S3). We also investigated three GTKO-F1 pigs: Nos. 2216 (♂), 2217 (♂), and 2218 (♀), which were produced by the mating of GTKO-F0 pig Nos. 669 (♂) and 657 (♀) (Figure S1B; Table S3). We validated the genotypes of the cell lines and GTKO pigs using Sanger sequencing at the DNA level, as well as fluorescence-activated cell sorting (FACS) and Western blotting at the protein level (Figures S13 and S14). We performed WGS of the ear samples from five GTKO-F0 pigs (Nos. 666, 669, 657, 659, and 681) and the two original WT pigs (Nos. 153 and 214) with a mean depth of 110× across the entire *Sus scrofa* 11.1 reference genome (Table S9) on the DNBSEQ-T7 platform, with sequencing quality, uniformity of coverage, percent GC coverage, and variant accuracy comparable to those of Illumina sequencing platforms [70]. To study how the genetic mutations in GTKO-F0 pigs arose, we also performed WGS on the WT cell lines (named WT-153-C and WT-214-C) and the GTKO cell lines [named KO-153-C(−2/+1), KO-214-C(−2/+1), and KO-214-C(−1/+1)] with an average depth of 110× across the entire *Sus scrofa* 11.1 reference genome (Table S9) on the DNBSEQ-T7 platform. The WGS data exhibited high read quality with Q30 > 90%, duplicate percentage < 6%, and properly paired reads > 98% (Table S9). A dendrogram of identity-by-state (IBS) distance verified the parentage of the 15 sequenced genomes among the DNA sequences involved in this study (Figure 1D). Genome sequences derived from the same pig were closely related and clustered together. The identity-by-descent (IBD) values also confirmed the genetic relationships between the GTKO-F1 pigs (Nos. 2216, 2217, and 2218) and their parents (GTKO-F0 pig Nos. 669 and 657) (Figure S15).

### CRISPR/Cas9 sgRNA design and plasmid construction

sgRNA was designed to target *GGTA1* (sgRNA sequence: 5′-GAGAAAATAATGAATGTCAA-3′) using the optimized CRISPR design tool (http://crispr.mit.edu/) and synthesized by Beijing Genomics Institution (GBI; Guangzhou, China). The sgRNAs were annealed to complementary oligos to generate double-stranded DNA and then cloned into the PX458-Cas9 plasmid (http://www.addgene.org/48138/sequences/). The *GGTA1* gene structure was downloaded from the University of California Santa Cruz (UCSC) Genome Browser (https://genome.ucsc.edu).

### Cell culture

Ear tissue samples were collected from Chinese Wuzhishan pigs and soaked in 75% ethanol, and the surface hair, cartilage, and fat were removed. The epidermal tissue was retained and cultured in culture medium containing 20% fetal bovine serum (Catalog No. A5669701, Gibco, Waltham, MA) and 5% penicillin (Catalog No. 15140122, Gibco) for 1–6 days to obtain Chinese Wuzhishan pig ear fibroblasts. All cells were maintained in Dulbecco’s modified Eagle’s medium (DMEM; Catalog No. 12100046, Gibco) in a humidified tri-gas incubator at 37°C under 5% CO_2_, 90% N_2_, and 5% O_2_.

### GTKO cell production

The targeting plasmid (4 μg; Catalog No. VT000062, Solarbio Life Sciences, Beijing, China) was co-electroporated into 1 × 10^6^ suspended Chinese Wuzhishan minipig ear fibroblasts using an Amaxa electrotransfection instrument (Amaxa, Cologne, Germany). Among them, 5000 cells were sampled to test the mutation efficiency, and the remaining cells were seeded in 100-mm plates (100 cells/plate). Then, the cells were cultured for 12–15 days to obtain single colonies. Individual colonies were transferred into 48-well plates and grown to confluence. The GTKO genotypes of individual clones were confirmed by PCR and Sanger sequencing using primers for the *GGTA1* target site.

### SCNT and selection of GTKO pigs

The SCNT protocol followed Pan and colleagues [71]. The ear tissues of the piglets produced by SCNT were collected, and DNA was extracted for PCR using a ZR Genomic DNATM-Tissue MiniPrep kit (Catalog No. D3051, Zymo Research, Irvine, CA). Then, the PCR products were subjected to electrophoresis. Primer information is provided in Table S12. All Chinese Wuzhishan miniature pigs were fed freely in the ordinary-grade pig house of Chengdu Zhongkeouge Biological Co., Ltd., China.

### Validation of genetic modification at the protein level using FACS

Peripheral blood mononuclear cells (PBMCs) derived from GTKO-F0 pigs and WT pigs were isolated by whole-blood lysis and then stained with FITC-BSIB4. *GGTA1* inactivation was analyzed by assessing the protein level of α-Gal with fluorescence microscopy and flow cytometry.

### Health assessment

For health status evaluations, eight adult GTKO pigs were randomly selected to fast for 16 h, and their body weights were measured in the awake state. Whole blood from the anterior vena cava was collected for measurements of blood physiological and biochemical indices (Mindray BC-6800 and Beckman Coulter AU5841), including white blood cell count, red blood cell count, and levels of aspartate aminotransferase, alanine aminotransferase, alkaline phosphatase, total protein, uric acid, creatinine, and blood glucose. The results were compared with those of age-appropriate WT pigs. To assess the breeding performance, the litter number and live litter size of three consecutive litters of GTKO-F0 sows were counted and compared with those of the WT population. Prism 9 software was used to perform statistical analysis. Measurement data were presented as mean ± standard deviation. Differences among multiple groups were analyzed by analysis of variance, with *P* < 0.05 considered statistically significant.

### Prediction of off-target sites of the *GGTA1* sgRNA

The potential off-target sites of the *GGTA1* sgRNA with the “NGG” or “NAG” PAM motif across the *Sus scrofa* 11.1 reference genome (http://ftp.ensembl.org/pub/release-103/fasta/sus_scrofa/dna/) were identified using Cas-OFFinder (v3.0.0) [51] based on sequence homology to the *GGTA1* sgRNA, allowing up to 3 mismatches with 1-bp indel and up to 4 mismatches with no indels.

### DNA extraction, library preparation, and WGS

Genomic DNA was extracted from ear tissues of WT and GTKO pigs, fibroblasts, and cell lines using the S32-Magnetic Universal Genomic DNA Kit (Catalog No. DP705-03, TIANGEN, Beijing, China) according to the manufacturer’s instructions. DNA sequencing libraries were generated using the VAHTS Universal DNA Library Prep Kit for Illumina V3 (Catalog No. ND607, Vazyme Biotech, Nanjing, China). WGS was performed on the DNBSEQ-T7 platform.

On average, 300 Gigabases (Gb) of raw sequence data were obtained for each sample. Sequencing read quality was assessed using FastQC (v0.11.5) [72] and MultiQC (v1.11) [73]. Fastq files were split across different lanes on the sequencing instrument. The paired-end reads were then mapped to the *Sus scrofa* 11.1 reference genome using the BWA-MEM algorithm within the BWA software package (v0.7.17) [74]. The duplicate reads were masked with the MarkDuplicatesSpark tool in GATK (v4.1.8.0) [28]. The quality scores of sequencing reads were recalibrated with the BaseRecalibrator and ApplyBQSR tools in GATK (v4.1.8.0) [28] using a set of known variants (https://ftp.ncbi.nih.gov/snp/pre_build152/organisms/archive/pig_9823/VCF/).

Alignment statistical metrics were collected using Qualimap (v2.1.2) [75]. Only mutations located on autosomes were considered for subsequent analyses.

### Genetic distance assessment and kinship validation

All autosomal single-nucleotide polymorphisms (SNPs) for all sequenced genomes were jointly identified by GATK (v4.1.8.0) [28] using the best practices for germline short variant discovery specified in a previous study [76]. The IBS pairwise distance matrix was calculated with filtered variants using PLINK (v1.9) [77] with the following parameters: --maf 0.05 --geno 0.05 --indep 50 5 2 --distance 1-ibs. A cluster dendrogram representing genetic distance was produced using the hclust function in R. The IBD results for each offspring were obtained using PLINK (v1.9) [77].

### Calling of DNMs accumulated in GTKO-F0 pigs

To dissect how genetic mutations accumulated from original WT pigs to GTKO-F0 pigs, we first identified all DNMs accumulated in GTKO-F0 pigs and then determined the specific steps at which these mutations occurred in the process of producing GTKO-F0 pigs according to the genome sequence information of cell lines.

SNVs and indels accumulated in GTKO-F0 pigs relative to the original WT pigs were identified using three callers: Mutect2 in GATK [28], Strelka2 (v2.9.10) [29], and VarScan2 (v2.4.2) [30]. Read depth was estimated by Strelka2 [29]. To avoid high false-positive rates in individual callers, a serial variant filtering procedure was performed with the following criteria: (1) candidate mutations identified by Mutect2 were filtered using FilterMutectCalls in GATK [28]; (2) mutation quality was evaluated with “PASS” using Strelka2 [29]; (3) mutation quality was evaluated with “high confidence” by VarScan2 [30]; (4) overlapping the variant sets identified by three mutation callers above; (5) no variant-supporting reads in the control sample (to ensure true DNMs); (6) read depth ≥ 30; (7) 0.3 ≤ VAF ≤ 0.7 (to eliminate mosaic mutations in GTKO-F0 pigs); and (8) manual checking of the candidate mutations with the Integrative Genomics Viewer (IGV) tool [78]. To assign these DNMs accumulated in the corresponding GTKO-F0 pigs to specific steps of the process, candidate mutations between cell lines or between cell lines and bulk samples in the GTKO-F0 pig production process were called by Strelka2 [29]. Notably, mutations fixed in GTKO-F0 pigs may have occurred in cell lines at a low frequency. All DNMs in GTKO-F0 pigs were assigned to specific steps based on the presence of candidate mutations called by Strelka2 [29] and variant-supporting reads in cell lines.

*De novo* SVs (≥ 50 bp; deletions + duplications + insertions + inversions) in the GTKO-F0 pigs relative to the original WT pigs were identified by two callers: DELLY (v0.8.1) [37] and Manta (v1.6.0) [38]. Filtering included the following criteria: (1) SV quality evaluated with “PASS”; (2) SVs with precise breakpoints; (3) min size > 50 bp; (4) no mutation-supporting reads in the control sample and percentage of variant-supporting reads > 0.2; (5) SVs identified by both DELLY [37] and Manta [38] (overlapped with SURVIVOR software); and (6) manual checking of the candidate *de novo* SVs using the IGV tool. To assign the *de novo* SVs accumulated in corresponding GTKO-F0 pigs to specific steps of the mutagenesis process, candidate *de novo* SVs between cell lines or between cell lines and bulk samples in the GTKO-F0 pig production process were called by DELLY [37] and Manta [38]. All *de novo* SVs in GTKO-F0 pigs were assigned to specific cell lines according to the presence of candidate *de novo* SVs and manual inspection by IGV.

### Trio calling of DNMs in GTKO-F1 pigs

Short variants including SNPs and indels were called using HaplotypeCaller in GATK [28], Platypus (v0.1.5) [42], Freebayes (v1.3.6) [43], and SAMtools (v1.3) [44] for each offspring and its parents, respectively. Variant filtering, referred to previously published filtering strategies [22], was performed on candidate variants. Briefly, site filtering and genotype filtering were performed to obtain high-confidence variants. Then, the DNMs for each offspring were identified using TrioDeNovo [45] with the default parameters and filtered according to the following criteria: (1) the DNM must have at least one allele absent from the parental genotypes, to avoid false positives arising from low-allele-fraction mosaic variants in either parent; (2) the DNM must be absent from the public pig variant database (dbSNPBuildID = 150); (3) the DNM must be called by all four different tools (considered high-confidence); and (4) every DNM locus was visually inspected in IGV to check whether any position was actually mosaic.

*De novo* SVs were called using DELLY [37] with the parameter “-q 20” and Manta [38] with default parameters for each offspring and its parents, respectively. Candidate *de novo* SVs called by DELLY [37] were filtered using the following criteria: (1) SV quality evaluated with “PASS”; (2) all samples were genotyped; (3) SVs with precise breakpoints; (4) min size > 50 bp; and (5) inconsistent with Mendelian inheritance. Candidate *de novo* SVs called by Manta [38] were filtered using the following criteria: (1) filtered by the filter mode of DELLY [37] with default parameters; (2) SVs with precise breakpoints; and (3) inconsistent with Mendelian inheritance. To obtain the final set of high-confidence SVs, *de novo* SV sets called by DELLY [37] and Manta [38] were merged using SURVIVOR [79] with the following criteria: (1) maximum allowed distance of 1 kb; (2) same SV type and on the same strand; and (3) manual checking of the *de novo* SVs using the IGV tool.

### Detection power for DNMs

We employed a method from Iyer et al. [41] to calculate the power of our WGS data for detecting mosaic DNMs. Given the median depth of 110× of our WGS data (Table S8) and a *de novo* variant allele frequency threshold of 10%, a heterozygous mutation in the two-cell embryo was expected to be observed in 27 mutant reads. Using the R function ppois (10, 27, lower.tail = TRUE), the probability of failing to call such a DNM (*i.e.*, observing ≤ 10 supporting reads) is 0.0002. This corresponds to the power of ≥ 99.98% for detecting DNMs occurring at the two-cell embryo stage (or earlier).

### DNM calling between treated and control cell lines

*De novo* SNV, indel, and SV calling between treated and control cell lines was performed, using a pipeline similar to that applied for DNM calling between GTKO pigs and WT pigs mentioned above. However, the filtering criteria were modified to require VAF ≤ 0.7.

### Functional effect prediction of mutations

For all identified mutations, annotation and functional effect prediction were performed based on sequence changes at the transcript level and amino acid changes at the protein level using SnpEff (v5.0e) [31] with the corresponding ENSEMBL gene annotation gtf file (release 99). The putative impacts of mutations were categorized as “modifier”, “low”, “moderate”, and “high” by SnpEff [31]. High-effect mutations were selected to assess the corresponding genome risk of GTKO pigs.

### Signature extraction from *de novo* SNVs in treated cell lines

Signature extraction of *de novo* SNVs in all treated cell lines was performed using nonnegative matrix factorization (NMF) with the R package MutationalPatterns (v3.4.0) [80] and the function “extract_signatures” with the following settings: rank = 2, nrun = 200. The cosine similarity of the extracted signature was refitted to the signatures of the COSMIC database [32].

### Assessment of plasmid sequence presence

The PX458 plasmid sequence used in our study was downloaded from http://www.addgene.org/48138/sequences/. Short-read sequencing data were aligned to both the reference genome and the plasmid sequence using BWA [74] with default parameters. The resulting alignments were then visually inspected using IGV. Read coverage across the plasmid sequence was calculated using the depth module in SAMtools [44] and plotted using Python in the Jupyter notebook.

To further confirm the insertion of the Cas9 plasmid by PCR, we used three primer pairs targeting GFP, Cas9, and ampicillin (AMP), which were positive selection markers. The primer sequences were as follows: GFP-F, 5′-CACATGAAGCAGCACGACTT-3′; GFP-R, 5′-TTCTCGTTGGGGTCTTTGCT-3′; Cas9-F, 5′-TCATATGCCAAGTACGCCCC-3′; Cas9-R, 5′-GGGCTCCGATCAGGTTCTTC-3′; AMP-F, 5′-CATCACCGAAACGCGCGAGA-3′; AMP-R, 5′-CAAGCAGCAGATTACGCGCAG-3′.

### Assessment of chromosome loss by read depth-based analysis

Coverage across the reference genome sequence was calculated using Qualimap with 400 windows. Figures were drawn using Python in the Jupyter notebook.

### Assessment of MSI

MSIsensor [40], a program that reports the percentage of unstable microsatellites as a score, was used to infer MSI status with paired sample mode. A cutoff MSISensor [40] score of 3.5 was used to discriminate MSI from MSS.

### Bulk RNA extraction, library preparation, and transcriptomic analysis

RNA was extracted from the blood of WT and GTKO pigs with TRIzol (Catalog No. 15596026, Thermo Fisher Scientific, Waltham, MA), and RNA-seq libraries were generated using the Hieff NGS MaxUp II Dual-mode mRNA Library Prep Kit (Catalog No. 12341ES, Yeasen Biotechnology, Shanghai, China). Libraries were sequenced on the DNBSEQ-T7 platform.

Sequencing read quality was assessed using FastQC (v0.11.5) [72] and MultiQC (v1.11) [72]. RNA-seq reads were aligned to the *Sus scrofa* 11.1 reference genome using STAR (v2.7.9a) [81] with the corresponding ENSEMBL gene annotation gtf file (release 103). Statistical analysis was performed in R (v.4.0.5). The average percentage of uniquely mapped reads was > 90% (Table S8). To investigate the expression of PERV, processed RNA-seq reads were also aligned to the PERV env sequences obtained from the National Center for Biotechnology Information (NCBI) GenBank (PERV-A: AF435966; PERV-B: AY099324; PERV-C: AY534304) using STAR [81] with the following parameters: --outSAMmultNmax 1 --alignIntronMax 1 --alignIntronMin 2 --scoreDelOpen --10000 --scoreInsOpen -10000. Read counts per gene locus were collected using the featureCount module in the R package Rsubread [82]. Normalization and differential gene expression analysis were performed using the R package DESeq2 (v.1.30.1) [83]. A heatmap was generated using the R package pheatmap (v1.0.12). Pearson correlation was calculated using the cor function in R, and figures were plotted using ggplot2 (v3.3.3) [84]. Genes with |log_2_ fold change| > 1 and a Benjamini–Hochberg-adjusted *P* value < 0.01 were considered significantly differentially expressed. GO and KEGG pathway enrichment analyses were performed using the web-based software DAVID (v2021; https://david.ncifcrf.gov/) [31].

### Confirmation of on-target mutations using WGS and RNA-seq

For WGS, read alignments at the *GGTA1* target site were examined manually in IGV to confirm the presence and zygosity of the expected genome edits. For RNA-seq, reads mapping to the *GGTA1* transcript were inspected in IGV to verify that the edited alleles were also reflected at the transcript level.

## Ethical statement

This study was approved by the Animal Experiments and Experimental Animal Welfare Committee of Capital Medical University, China (Approval No. AEEI-2020-001).

## Code availability

The source code has been submitted to BioCode at the National Genomics Data Center (NGDC), China National Center for Bioinformation (CNCB) (BioCode: BT007804), which is publicly accessible at https://ngdc.cncb.ac.cn/biocode/tool/7804. It is also available on the GitHub website (https://github.com/U201412486/GTKO_pigs).

## Supplementary Material

qzaf071_Supplementary_Data

## Data Availability

The sequencing data generated in this study have been deposited in the NCBI Sequence Read Archive (SRA: PRJNA915800), and are publicly accessible at https://www.ncbi.nlm.nih.gov/bioproject/PRJNA915800/. The data have also been deposited in the Genome Sequence Archive [85] at the NGDC, CNCB (GSA: CRA017838), and are publicly accessible at https://ngdc.cncb.ac.cn/gsa/search?searchTerm=CRA017838.

## CRediT author statement

**Xueyun Huo:** Methodology, Investigation, Visualization, Writing – original draft, Writing – review & editing. **Xianhui Sun:** Methodology, Investigation, Formal analysis, Visualization, Writing – original draft, Writing – review & editing. **Xiangyang Xing:** Methodology, Investigation, Visualization, Writing – original draft, Writing – review & editing. **Jing Lu:** Writing – review & editing. **Jingjing Zhang:** Investigation, Writing – review & editing. **Yanyan Jiang:** Methodology, Investigation, Writing – review & editing. **Xiao Zhu:** Writing – review & editing. **Changlong Li:** Writing – review & editing. **Jianyi Lv:** Writing – review & editing. **Meng Guo:** Writing – review & editing. **Lixue Cao:** Writing – review & editing. **Xin Liu:** Writing – review & editing. **Zhenwen Chen:** Conceptualization, Supervision. **Dengke Pan:** Conceptualization, Supervision. **Shunmin He:** Conceptualization, Supervision. **Chen Zhang:** Conceptualization. **Xiaoyan Du:** Conceptualization, Supervision, Writing – review & editing. All authors have read and approved the final manuscript.

## ORCID

0000-0002-1646-0963 (Xueyun Huo)

0009-0009-1408-566X (Xianhui Sun)

0000-0001-7275-9401 (Xiangyang Xing)

0009-0004-6370-2445 (Jing Lu)

0009-0008-8916-551X (Jingjing Zhang)

0009-0005-0619-0014 (Yanyan Jiang)

0009-0009-1416-849X (Xiao Zhu)

0000-0001-6832-785X (Changlong Li)

0000-0002-5288-5328 (Jianyi Lv)

0000-0002-1482-1446 (Meng Guo)

0000-0001-7640-2872 (Lixue Cao)

0000-0003-1337-6122 (Xin Liu)

0000-0003-4678-3437 (Zhenwen Chen)

0000-0002-7994-1465 (Dengke Pan)

0000-0002-7294-0865 (Shunmin He)

0000-0002-7940-8054 (Chen Zhang)

0000-0002-4030-7299 (Xianyan Du)
