# Supplementary material for: Genome-wide Genetic Mutations Accumulated in Pigs Genome-edited for Xenotransplantation and Their Filial Generation
Source: Genomics Proteomics Bioinformatics. 2025 Aug 20;23(4):qzaf071. doi: 10.1093/gpbjnl/qzaf071 (PMC12771377; doi:10.1093/gpbjnl/qzaf071)
Supplement: qzaf071_Supplementary_Data [file qzaf071_supplementary_data.zip › Figure S11.pdf]

A

WGS Reads(supported reads/total reads)

		153	WT-153-C	
WT：	CCTTTTCTTTTCCCAGGAGAAAATAATGAATGTCAA AGGAAGAGTGGTTCTGTCAA			
Allele 1/2：	CCTTTTCTTTTCCCAGGAGAAAATAATGAATGTCAAAGGAAGAGTGGTTCTGTCAA	123/123	129/129	
		KO-153-C(-2/+1)	666	669(sire)
Allele 1：-2	CCTTTTCTTTTCCCAGGAGAAAATAATGAAT ·· CAAAGGAAGAGTGGTTCTGTCAA	69/127	61/121	74/141
Allele 2：+1	CCTTTTCTTTTCCCAGGAGAAAATAATGAATGTTCAA AGGAAGAGTGGTTCTGTCAA	58/127	60/121	67/141
		214	WT-214-C	
WT：	CCTTTTCTTTTCCCAGGAGAAAATAATGAATGTCAAAGGAAGAGTGGTTCTGTCAA			
Allele 1/2：	CCTTTTCTTTTCCCAGGAGAAAATAATGAATGTCAAAGGAAGAGTGGTTCTGTCAA	103/103	154/154	
		KO-214-C(-1/+1)	681	
Allele 1：-1	CCTTTTCTTTTCCCAGGAGAAAATAATGAATGT·AAAGGAAGAGTGGTTCTGTCAA	63/133	45/100	
Allele 2：+1	CCTTTTCTTTTCCCAGGAGAAAATAATGAATGTTCAAAGGAAGAGTGGTTCTGTCAA	70/133	55/100	
		KO-214-C(-2/+1)	657(dam)	659
Allele 1：+1	CCTTTTCTTTTCCCAG GAGAAAATAATGAATGTTCAAAGGAAGAGTGGTTCTGTCAA	53/102	65/129	51/118
Allele 2： -2	CCTTTTCTTTTCCCAGGAGAAAATAATGAATGTAG····GGAAGAGTGGTTCTGTCAA	49/102	64/129	57/118
Offspring		2216	2217	
Allele 1：+1	CCTTTTCTTTTCCCAGGAGAAAATAATGAATGTTCAAAGGAAGAGTGGTTCTGTCAA	50/114	58/126	
Allele 2：-2	CCTTTTCTTTTCCCAGGAGAAAATAATGAATGTAG···· GGAAGAGTGGTTCTGTCAA	64/114	68/126	
		2218		
Allele 1：-2	CCTTTTCTTTTCCCAGGAGAAAATAATGAAT ·· CAAAGGAAGAGTGGTTCTGTCAA	58/114		
Allele 2：+1	CCTTTTCTTTTCCCAGGAGAAAATAATGAATGTTCAAAGGAAGAGTGGTTCTGTCAA	56/114		

gRNA PAM

B

RNA Reads (supported reads/total reads)

		872	876	937	950
WT	GAGAAAATAATGAATGTCAA AGGAAGAGTGGTTCTGTCAA	164/164	242/242	154/154	164/164
Allele 1/2	GAGAAAATAATGAATGTCAA AGGAAGAGTGGTTCTGTCAA				
		657	659		
Allele 1：+1	GAGAAAATAATGAATGTTCAA AGGAAGAGTGGTTCTGTCAA	71/141	110/179		
Allele 2： -2	GAGAAAATAATGAATGTAG···· GGAAGAGTGGTTCTGTCAA	70/141	69/179		
		669			
Allele 1：-2	GAGAAAATAATGAAT ·· CAAAGGAAGAGTGGTTCTGTCAA	26/75			
Allele 2：+1	GAGAAAATAATGAATGTTCAA AGGAAGAGTGGTTCTGTCAA	49/75			
		2216	2217		
Allele 1：+1	GAGAAAATAATGAATGTTCAA AGGAAGAGTGGTTCTGTCAA	18/36	66/132		

gRNA PAM
