# Supplementary material for: Genome-wide Genetic Mutations Accumulated in Pigs Genome-edited for Xenotransplantation and Their Filial Generation
Source: Genomics Proteomics Bioinformatics. 2025 Aug 20;23(4):qzaf071. doi: 10.1093/gpbjnl/qzaf071 (PMC12771377; doi:10.1093/gpbjnl/qzaf071)
Supplement: qzaf071_Supplementary_Data [file qzaf071_supplementary_data.zip › Figure S13.pdf]

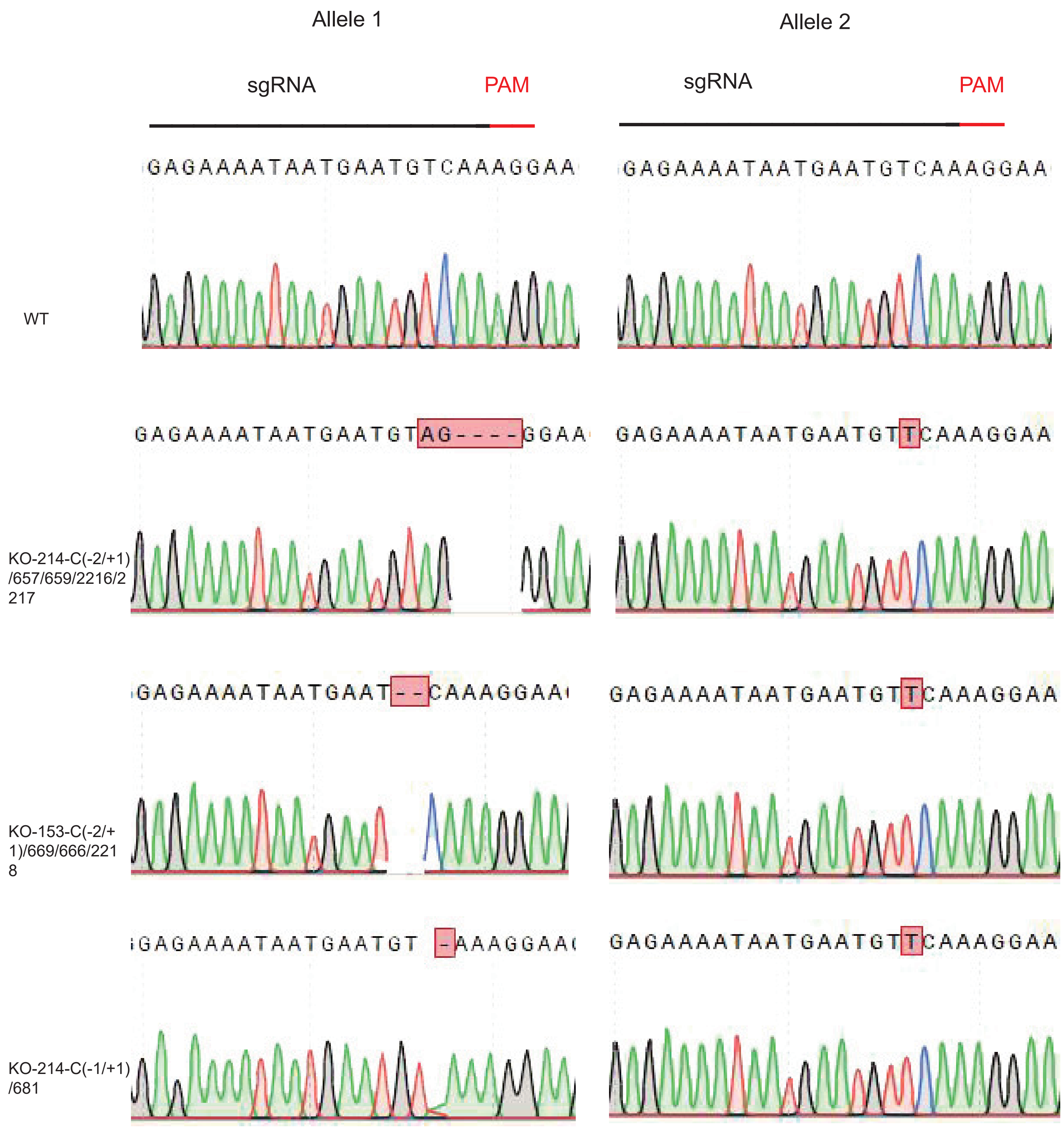

Allele 1
Allele 2
sgRNA
PAM
sgRNA
PAM
GAGAAAATAATGAATGTCAAAGGAA
GAGAAAATAATGAATGTCAAAGGAA
WT
GAGAAAATAATGAATGTAG----GGAA
GAGAAAATAATGAATGTTTCAAAGGAA
KO-214-C(-2/+1)/657/659/2216/2217
GAGAAAATAATGAAT--CAAAGGAA
GAGAAAATAATGAATGTTTCAAAGGAA
KO-153-C(-2/+1)/669/666/2218
GAGAAAATAATGAATGT -AAAGGAA
GAGAAAATAATGAATGTTTCAAAGGAA
KO-214-C(-1/+1)/681
