# Supplementary material for: Genome-wide Genetic Mutations Accumulated in Pigs Genome-edited for Xenotransplantation and Their Filial Generation
Source: Genomics Proteomics Bioinformatics. 2025 Aug 20;23(4):qzaf071. doi: 10.1093/gpbjnl/qzaf071 (PMC12771377; doi:10.1093/gpbjnl/qzaf071)
Supplement: qzaf071_Supplementary_Data [file qzaf071_supplementary_data.zip › Supplementary Material Captions.docx]

**Supplementary** **material**

**Figure S1 Comparison of weight and litter size between GTKO pigs and WT pigs**

Box plots showing the comparison of the weight (**A**) and litter size (**B**) between GTKO pigs and WT pigs. ns, no significance, Wilcoxon rank sum test.

**Figure S2 Genomic sequence alignment to the donor plasmid**

The short-read genomic sequences from all samples were aligned to the PX458 plasmid.

**Figure S3 Agarose gel analysis showing unexpected integrations of the sgRNA-Cas9 plasmid into the genomes of GTKO-F0 pigs 681, 682, 683, 684, and 685**

The five tested pigs were derived from the same GGTA1-edited cell line, KO-214-C(−1/+1). “−”, wild-type pig; “+”, PX458 plasmid. The resulting fragment sizes were 413 bp (GFP), 926 bp (Cas9), and 1286 bp (AMP).

**Figure S4 Distribution of *de novo* SNV types**

SNVs accumulated in (**A**) GTKO pigs and (**B**) different treated cell lines.

**Figure S5 Average read depth revealing no chromosome duplication and loss events**

**Figure S6 Venn diagram of germline mutations called using four different tools**

**Figure S7 Venn diagram of DNMs called using four different tools**

**Figure S8 RNA-seq read alignment to envelope genes (*env*) sequences of three PERV subtypes**

**Figure S9 PCA showing clustering of RNA-seq pigs by condition and gender**

PC1, explaining 29% of the total variance, separates F0/F1 pigs from WT pigs; PC2, explaining 19% of the total variance, seems to distinguish a sex-related factor.

**Figure S10 RNA-seq data analysis**

**A.** Heatmap showing the Pearson’s correlations of RNA-seq expression data between samples. Volcano plots illustrating 19 DEGs between WT pigs and GTKO-F0 pigs (**B**) and 215 DEGs between WT pigs and GTKO-F1 pigs (**C**). **D.** DAVID functional annotation showing GO and KEGG enrichment analysis of DEGs between WT pigs and GTKO-F1 pigs. The horizontal and vertical axes represent GO or KEGG term names and −Log_10_ *P* value of the corresponding terms, respectively.

**Figure S11 Allele read count data of WGS data and RNA-seq data showing on-target results**

**Figure S12 Circos plot displaying potential off-target sites of *GGTA1* sgRNA predicted by Cas-OFFinder**

**Figure S13 Sanger sequencing of GTKO cell lines and pigs at on-target sites**

**Figure S14 Validation of *GGTA1* on-target mutation in GTKO pigs at the protein level**

**A.** Expression of alpha-(1,3) Gal by fluorescence-activated cell sorting (FACS). **B.** Expression of alpha-(1,3) Gal by Western blotting.

**Figure S15 Trio genetic relationships**

Each cell indicates an IBD score calculated from individual pairs. Darker color indicates a higher IBD score and a closer relationship.

**Table S1 Statistics of blood physiology and blood chemistry**

**Table S2 Short mutations accumulated in CRISPR/Cas9 step and Nuclear/Transfer step of the pigs**

**Table S3 Information of Chinese Wuzhishan minipigs**

**Table S4 Statistics of SVs at each filter step**

**Table S5 Genome-wide microsatellite instability quantification using MSIsensor**

**Table S6 Statistics of identified high-confidence DNMs**

**Table S7 Statistics of variants (SVs) in the trio**

**Table S8 Quality control of RNA-seq data**

**Table S9 Quality control of WGS data**

**Table S10 Predicted off-target locations by Cas-OFFinder**

**Table S11 SNVs and indels accumulated in CRISPR/Cas9-treated porcine fibroblast cell lines**

**Table S12 Primers for on-target genotyping**

**Table S13 Targeting efficiency of sgRNA in Chinese Wuzhishan minipig fibroblasts**

**Table S14 SCNT success rate**
