# Supplementary material for: Genome-wide Genetic Mutations Accumulated in Pigs Genome-edited for Xenotransplantation and Their Filial Generation
Source: Genomics Proteomics Bioinformatics. 2025 Aug 20;23(4):qzaf071. doi: 10.1093/gpbjnl/qzaf071 (PMC12771377; doi:10.1093/gpbjnl/qzaf071)
Supplement: qzaf071_Supplementary_Data [file qzaf071_supplementary_data.zip › Table S5.docx]

**Table S5 Genome-wide microsatellite instability quantification using MSIsensor**

| **Control sample** | **Treated sample** | **MSIsensor’s score** | **MSI score** |
| --- | --- | --- | --- |
| WT-153-A | WT-153-C | 0 | < 3.5 |
| WT-153-C | KO-153-C(−2+1) | 0.02 |  |
| KO-153-C(−2+1) | 669 | 0.02 |  |
| KO-153-C(−2+1) | 666 | 0.02 |  |
| WT-214-A | WT-214-C | 0 |  |
| WT-214-C | KO-214-C(−2+1) | 0.02 |  |
| KO-214-C(−2+1) | 659 | 0.01 |  |
| KO-214-C(−2+1) | 657 | 0.01 |  |
| WT-214-C | KO-214-C(−1+1) | 0.02 |  |
| KO-214-C(−1+1) | 681 | 0.01 |  |
| WT-214-A | 657 | 0.06 |  |
| WT-214-A | 659 | 0.04 |  |
| WT-214-A | 681 | 0.05 |  |
| WT-153-A | 666 | 0.05 |  |
| WT-153-A | 669 | 0.08 |  |
