# Supplementary material for: Genome-wide Genetic Mutations Accumulated in Pigs Genome-edited for Xenotransplantation and Their Filial Generation
Source: Genomics Proteomics Bioinformatics. 2025 Aug 20;23(4):qzaf071. doi: 10.1093/gpbjnl/qzaf071 (PMC12771377; doi:10.1093/gpbjnl/qzaf071)
Supplement: qzaf071_Supplementary_Data [file qzaf071_supplementary_data.zip › Table S14.docx]

**Table S14 SCNT success rate**

| **Batch** | **Recipient sow** | **Clone** | **Number of reconstructed embryos** | **Piglet number** | **SCNT success rate** |
| --- | --- | --- | --- | --- | --- |
| 1 | 20-2(548) | 153-GTKO-A26 | 304 | 0 | 0% |
| 2 | 20-8(546) | 214-GTKO-A10 | 153 | 0 | 0% |
| 3 | 20-9(545) | 214-GTKO-A10 | 139 | 4 | 2.90% |
| 4 | 20-12(468) | 153-GTKO-A26 | 134 | 11 | 6.70% |
|  |  | 153-GTKO-A39 | 30 |  |  |
| 5 | 20-20(410) | 214-GTKO-A7 | 134 | 7 | 5.20% |
| 6 | 20-21(574) | 214-GTKO-A1 | 97 | 0 | 0% |
|  |  | 214-GTKO-A7 | 86 |  |  |
| **Total** |  |  | **1077** | **22** | **2.04%** |
