# Supplementary material for: Genome-wide Genetic Mutations Accumulated in Pigs Genome-edited for Xenotransplantation and Their Filial Generation
Source: Genomics Proteomics Bioinformatics. 2025 Aug 20;23(4):qzaf071. doi: 10.1093/gpbjnl/qzaf071 (PMC12771377; doi:10.1093/gpbjnl/qzaf071)
Supplement: qzaf071_Supplementary_Data [file qzaf071_supplementary_data.zip › Table S12.docx]

**Table S12 Primers for on-target genotyping**

| **GGTA1 sgRNA** | **Primer** | **Sequence (5′ to 3′)** |
| --- | --- | --- |
| GAGAAAATAATGAATGTCAAAGG | F1 | CGTTGCTGTCCGTGAGTTGT |
|  | R1 | TCTTTACGGTGTCAGTGAATCCTAC |
|  | F2 | TAAACCCGTCACTCTCCCAC |
|  | R2 | ACGGTGTCAGTGAATCCTAC |
