# Supplementary figures and images for: Genome-wide Genetic Mutations Accumulated in Pigs Genome-edited for Xenotransplantation and Their Filial Generation
Source: Genomics Proteomics Bioinformatics. 2025 Aug 20;23(4):qzaf071. doi: 10.1093/gpbjnl/qzaf071 (PMC12771377; doi:10.1093/gpbjnl/qzaf071)

A

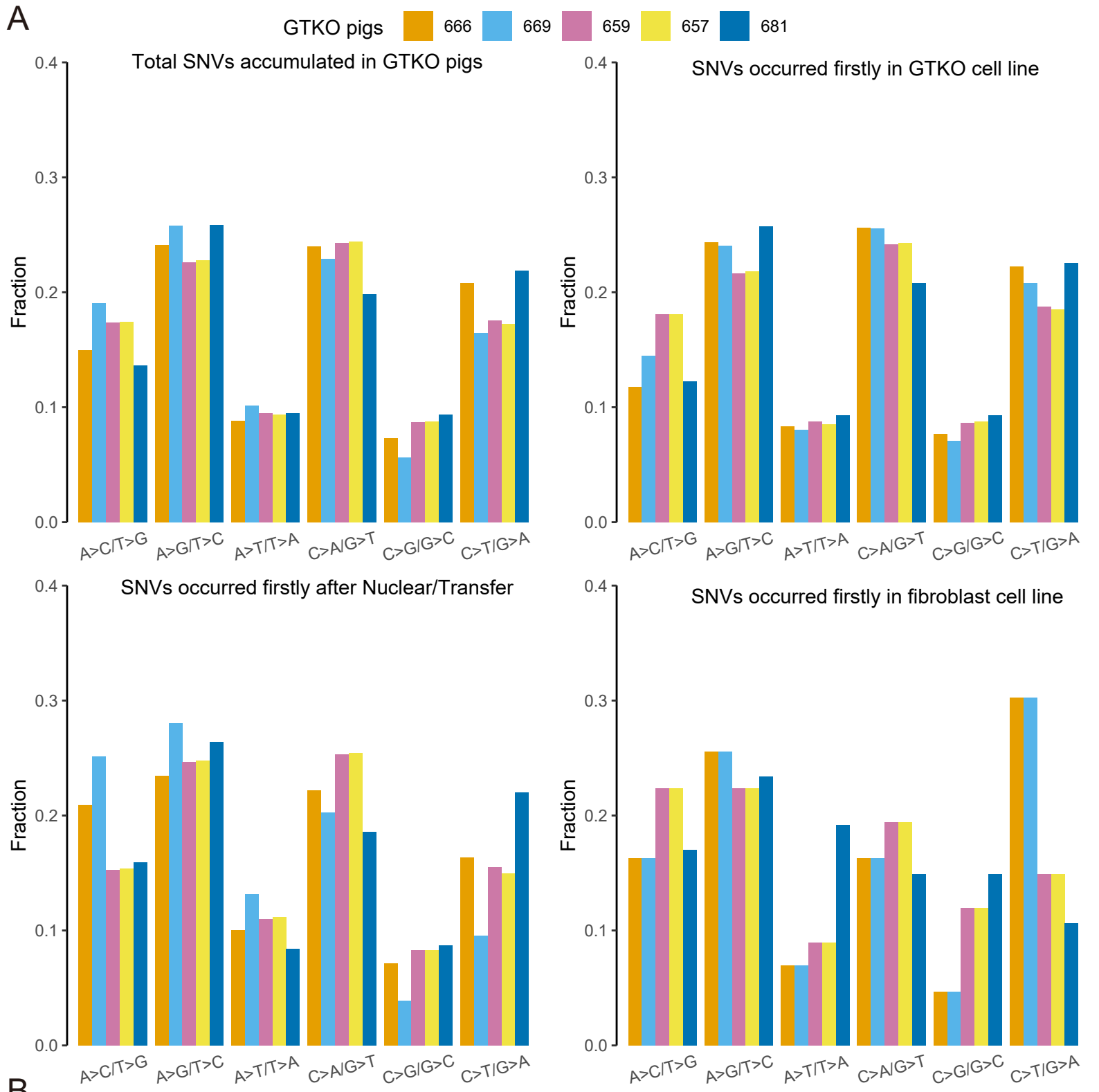

B

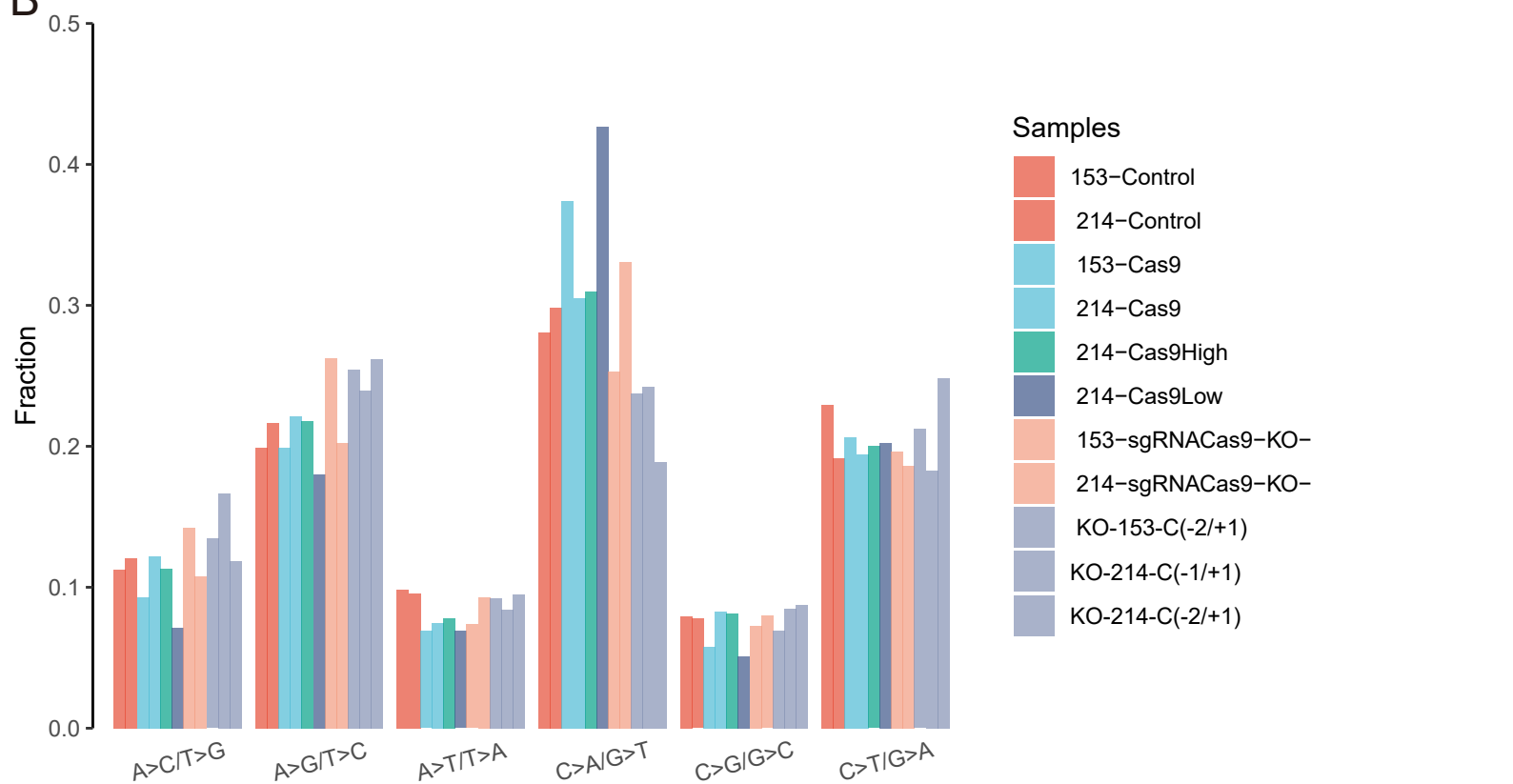

Supplement: qzaf071_Supplementary_Data [file qzaf071_supplementary_data.zip › Figure S4.pdf]

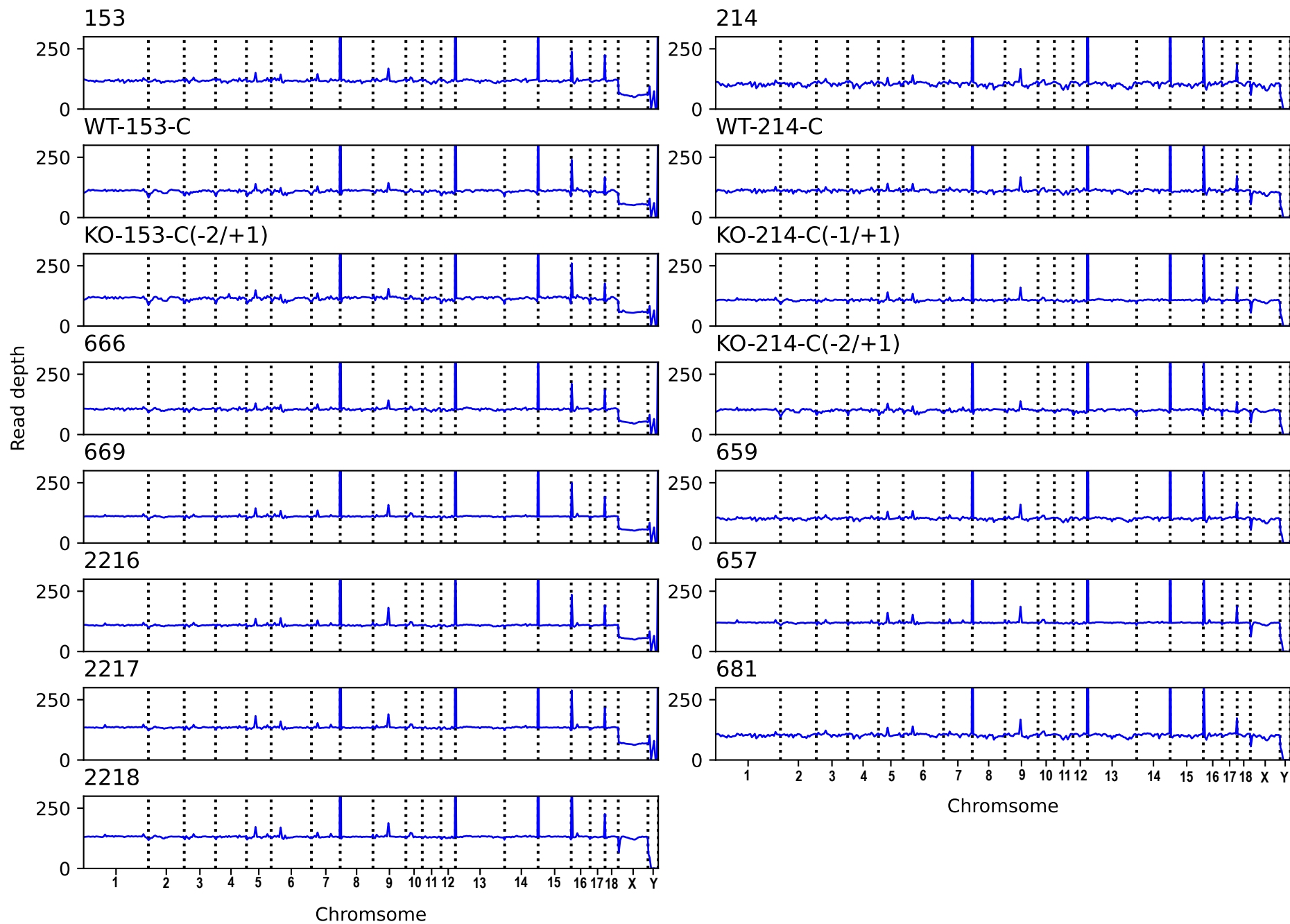

Supplement: qzaf071_Supplementary_Data [file qzaf071_supplementary_data.zip › Figure S5.pdf]

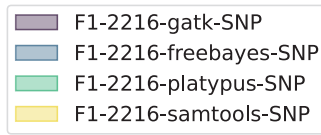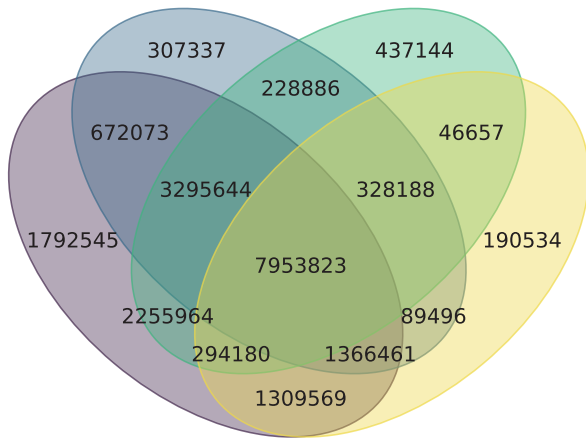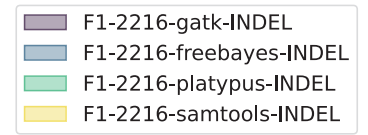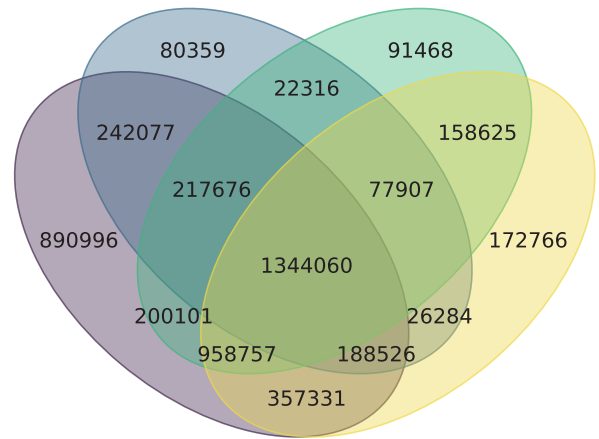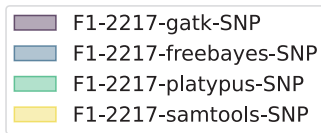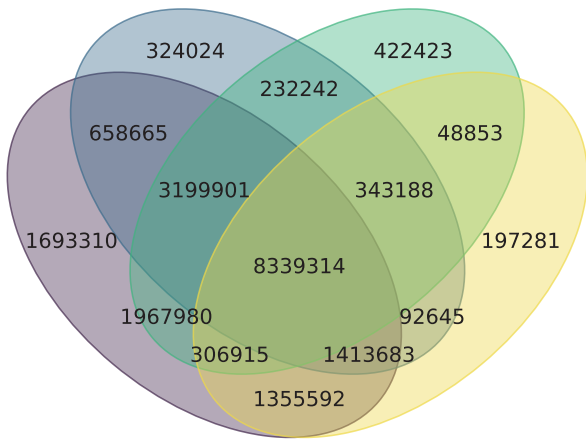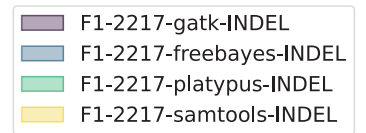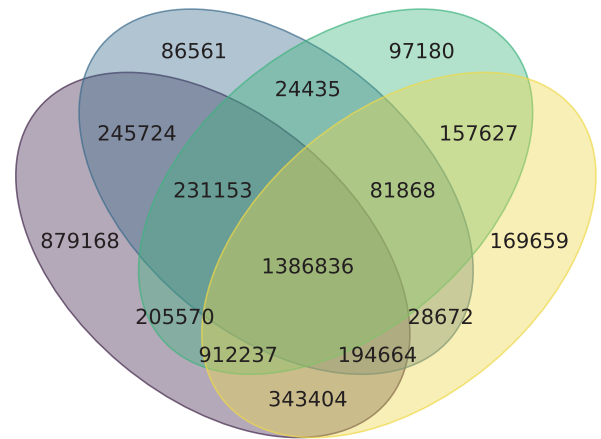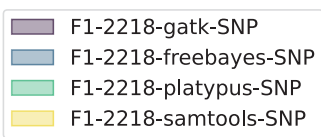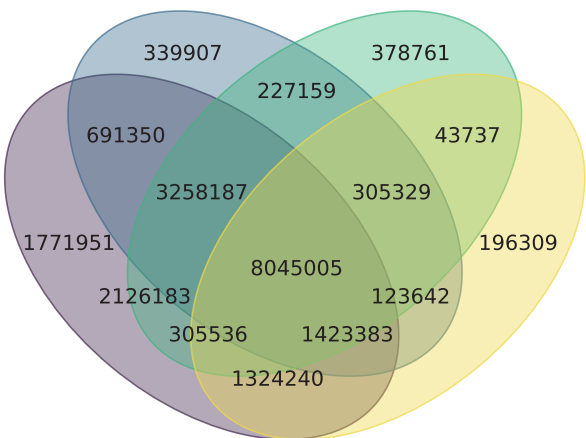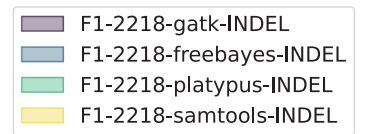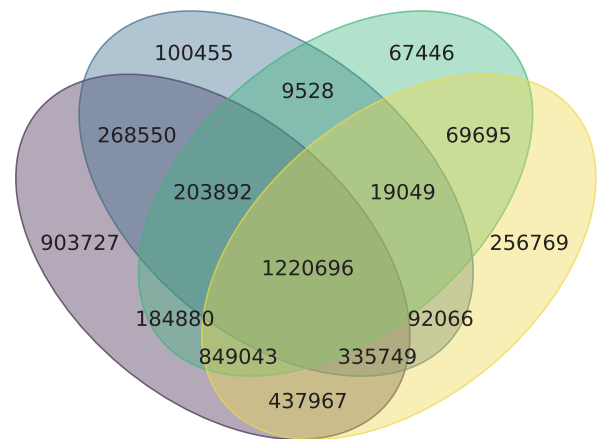

Supplement: qzaf071_Supplementary_Data [file qzaf071_supplementary_data.zip › Figure S6.pdf]

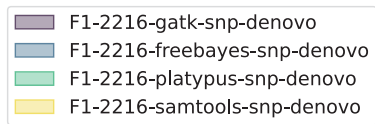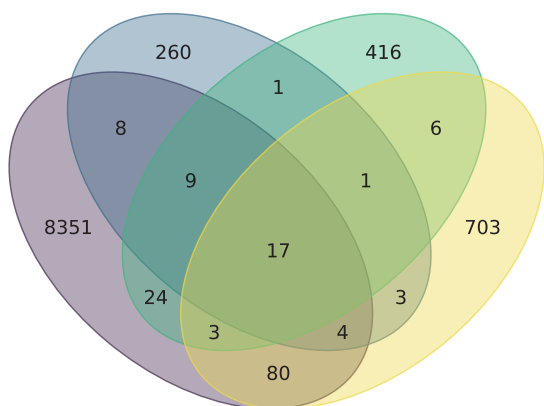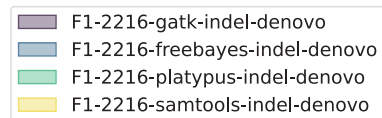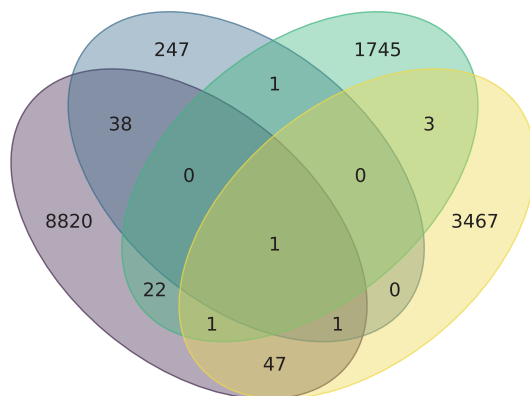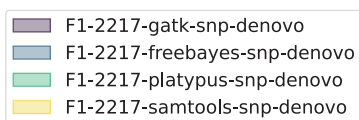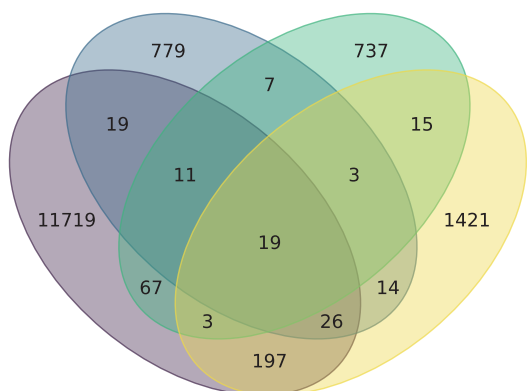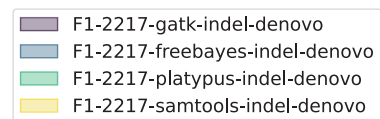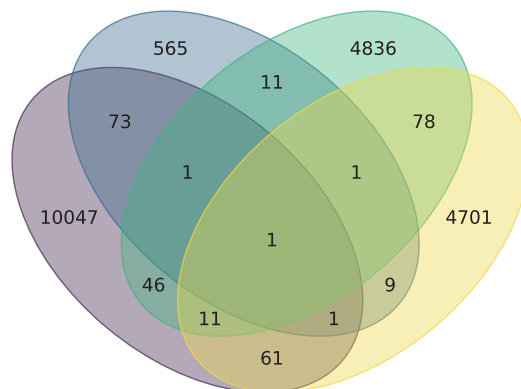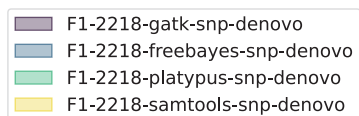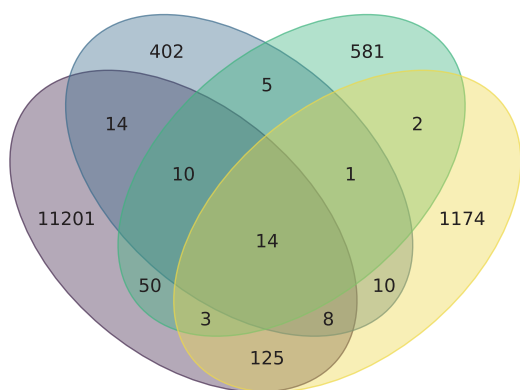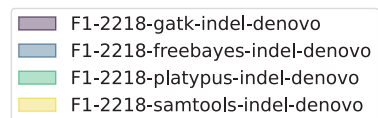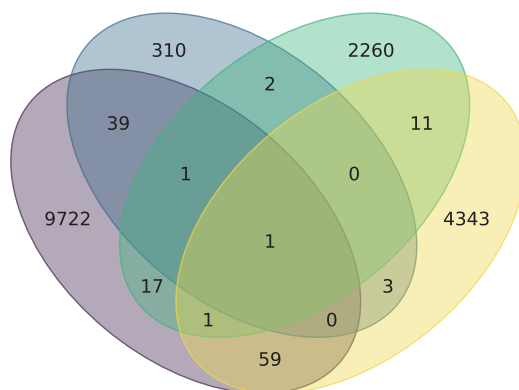

Supplement: qzaf071_Supplementary_Data [file qzaf071_supplementary_data.zip › Figure S7.pdf]

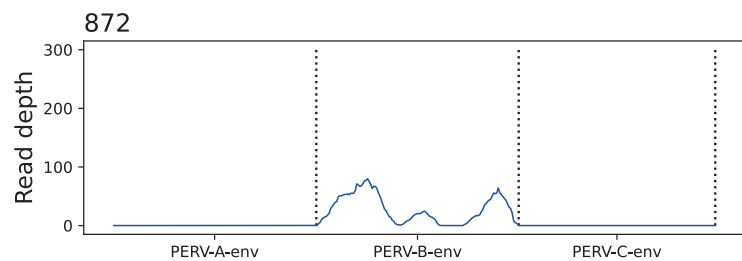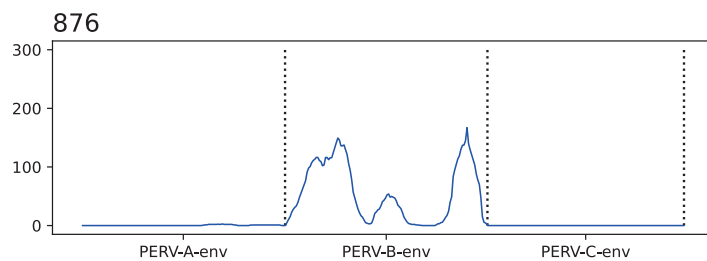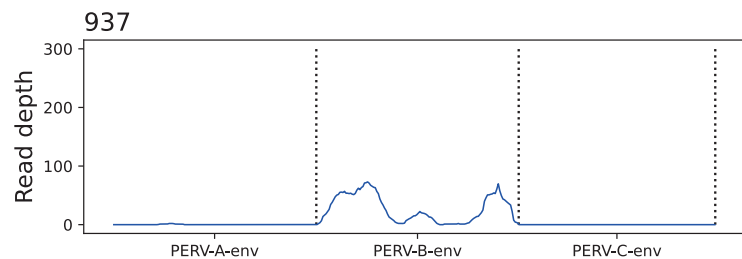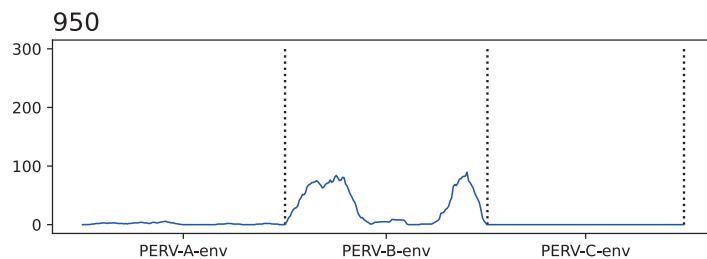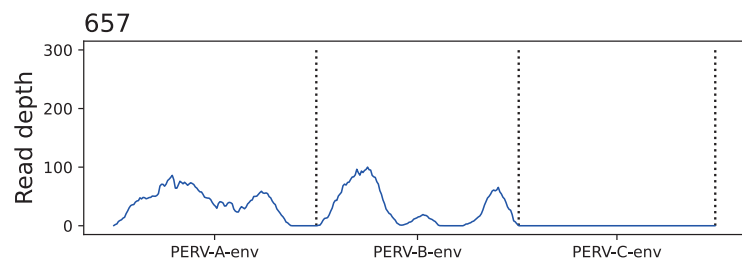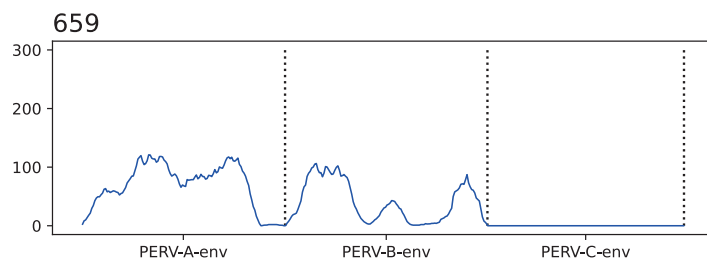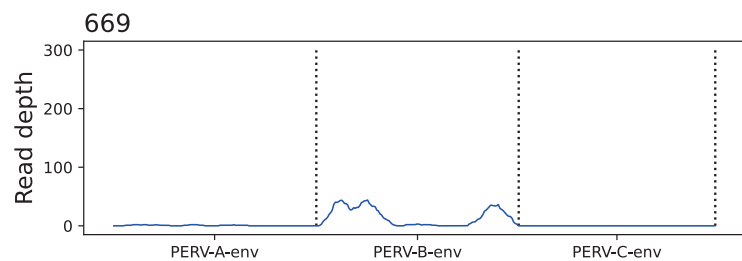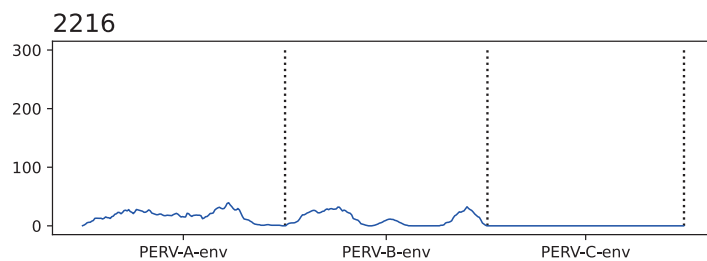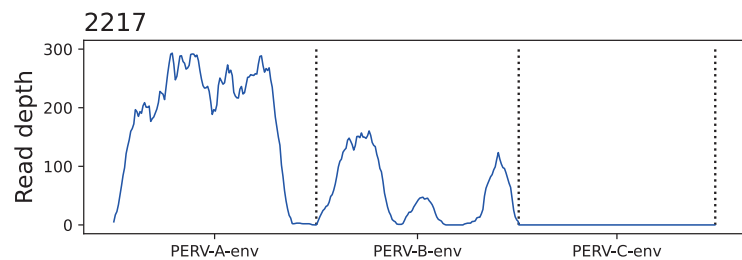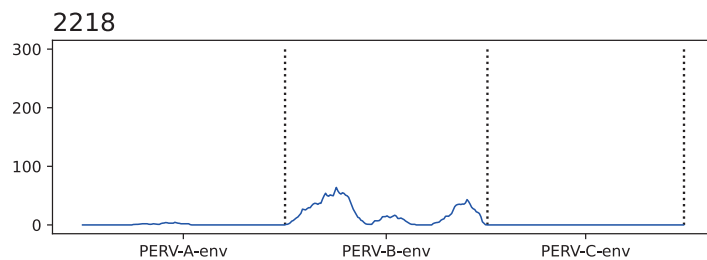

Supplement: qzaf071_Supplementary_Data [file qzaf071_supplementary_data.zip › Figure S8.pdf]

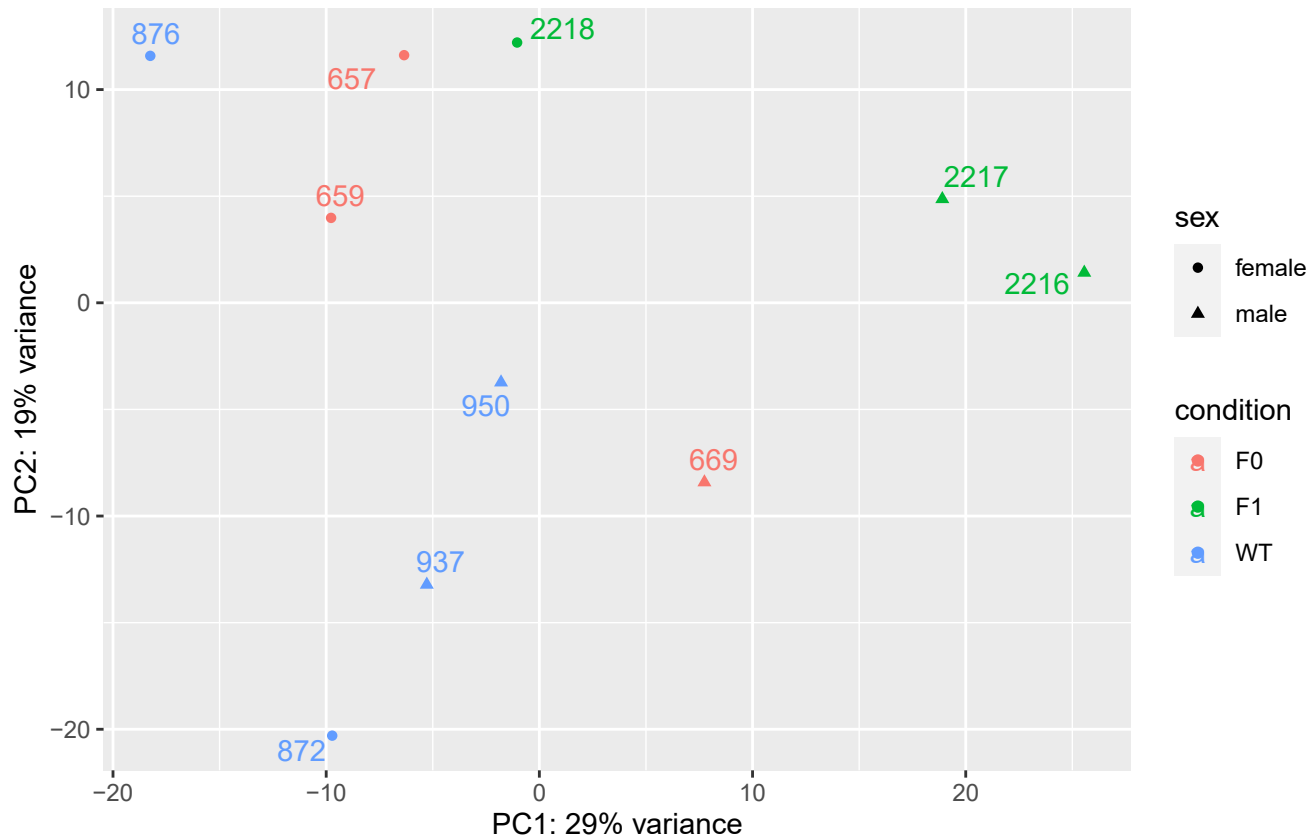

Supplement: qzaf071_Supplementary_Data [file qzaf071_supplementary_data.zip › Figure S9.pdf]

A

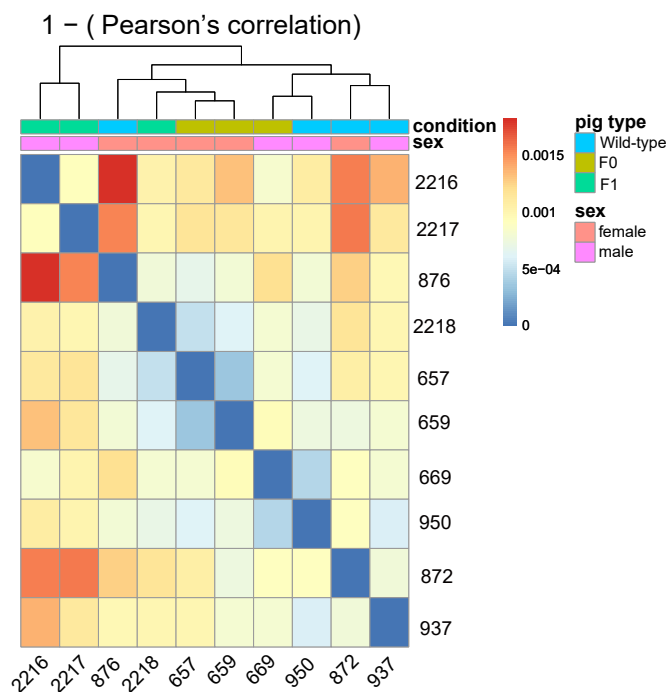

B

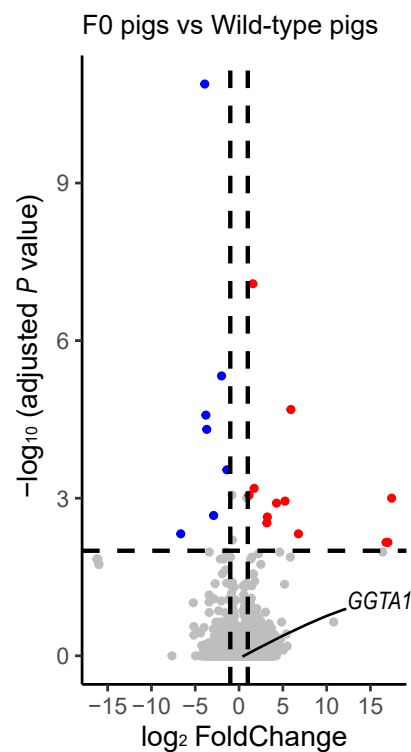

C

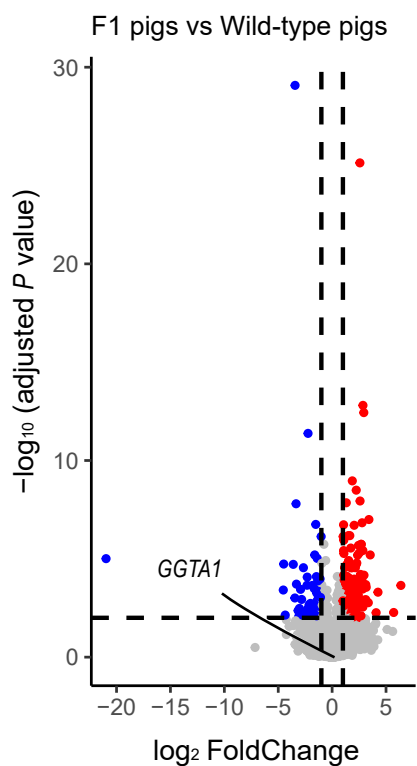

D

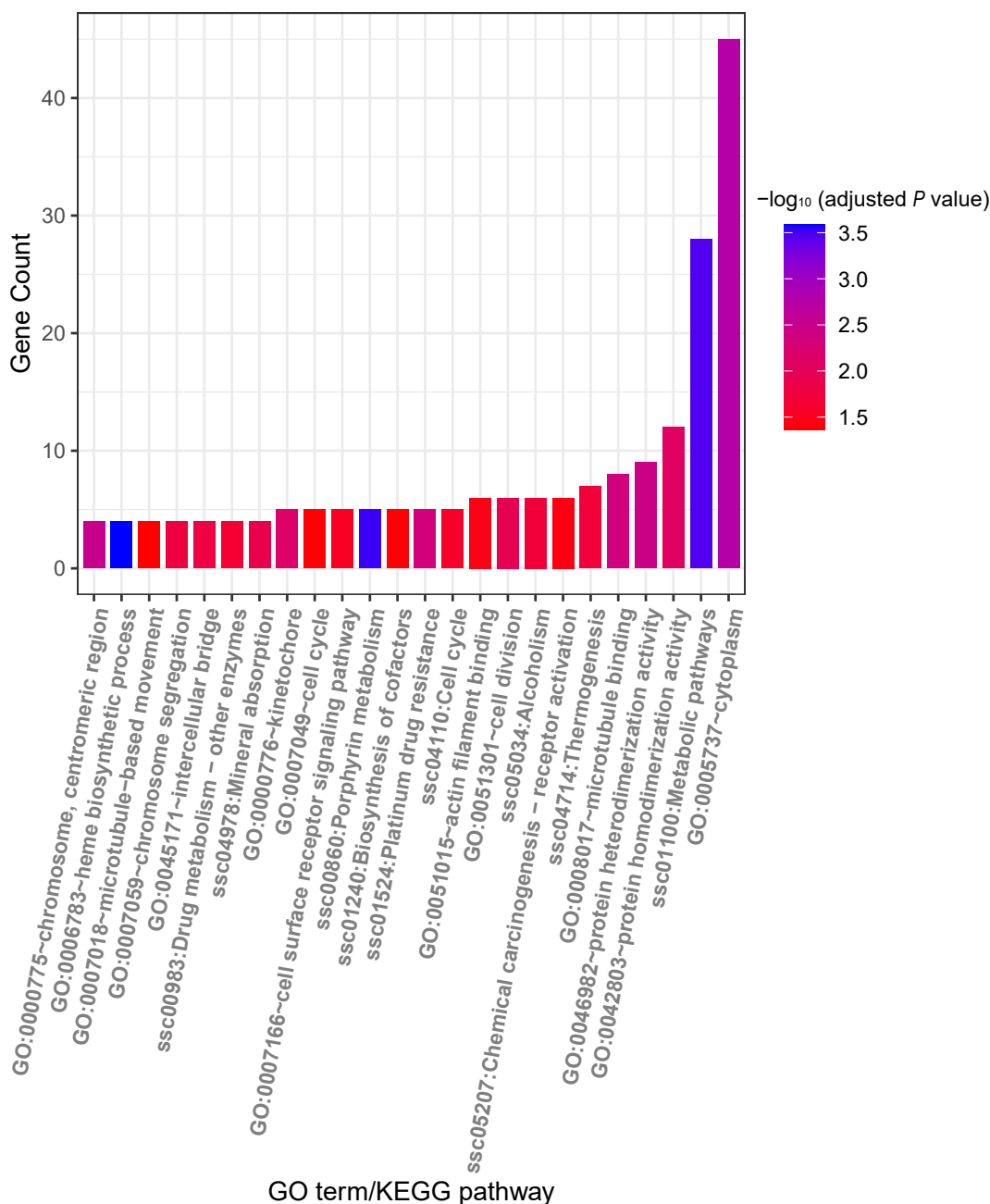

Supplement: qzaf071_Supplementary_Data [file qzaf071_supplementary_data.zip › Figure S10.pdf]

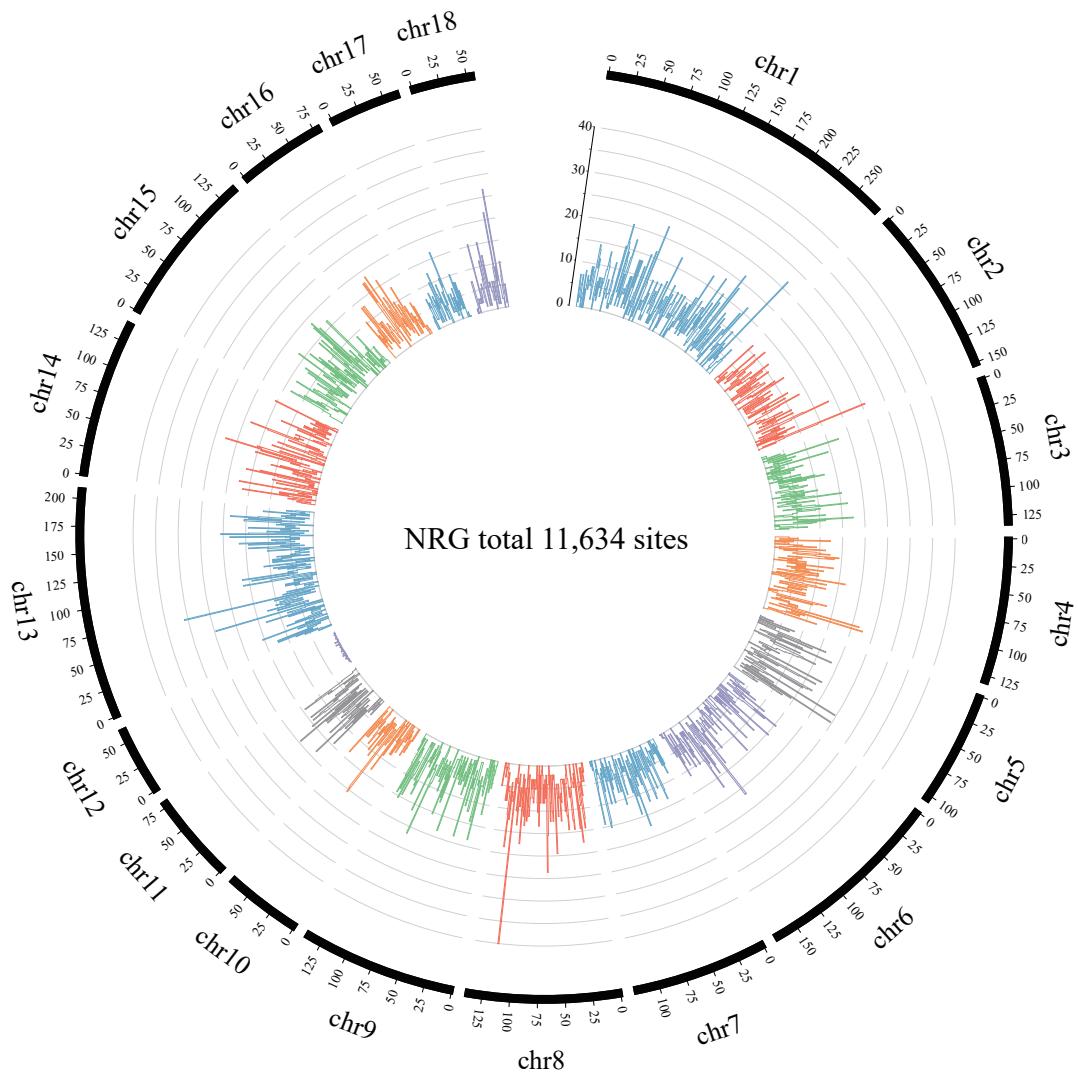

Supplement: qzaf071_Supplementary_Data [file qzaf071_supplementary_data.zip › Figure S12.pdf]

A

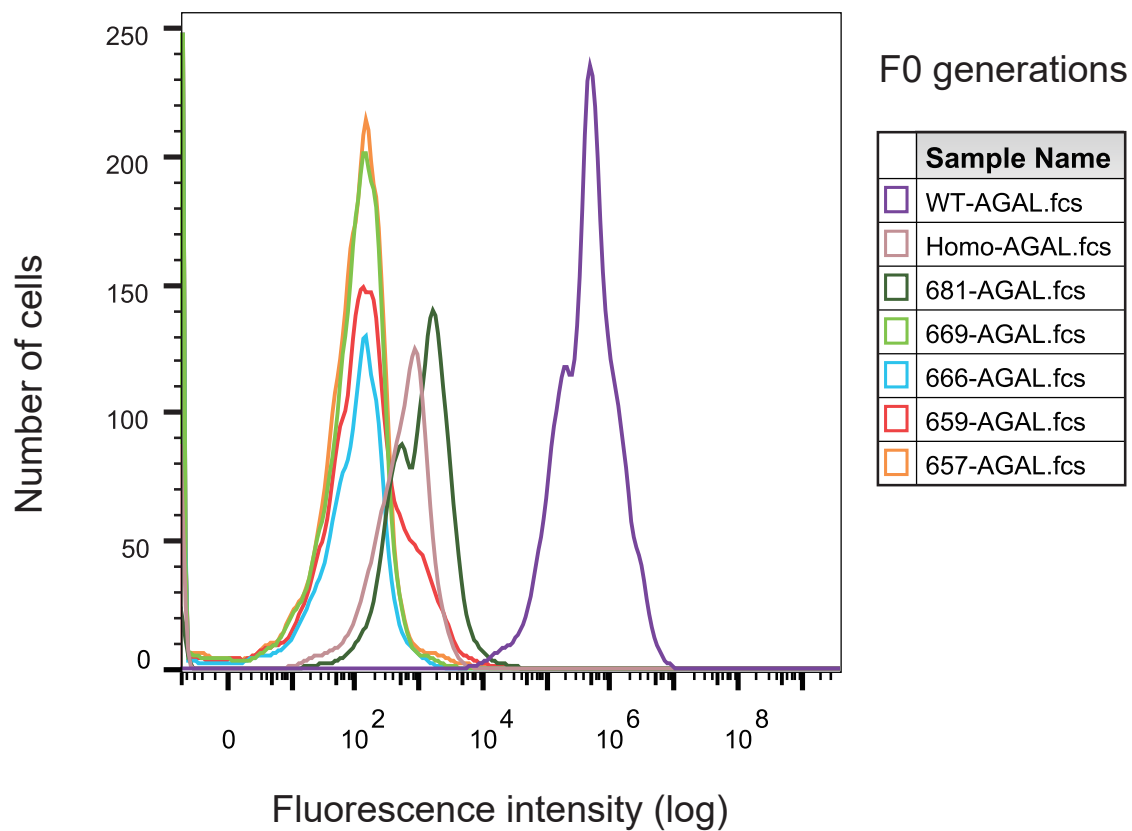

B

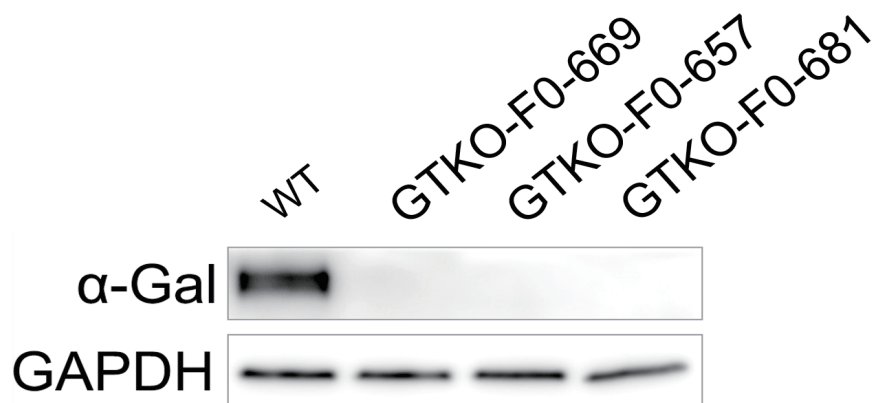

Supplement: qzaf071_Supplementary_Data [file qzaf071_supplementary_data.zip › Figure S14.pdf]

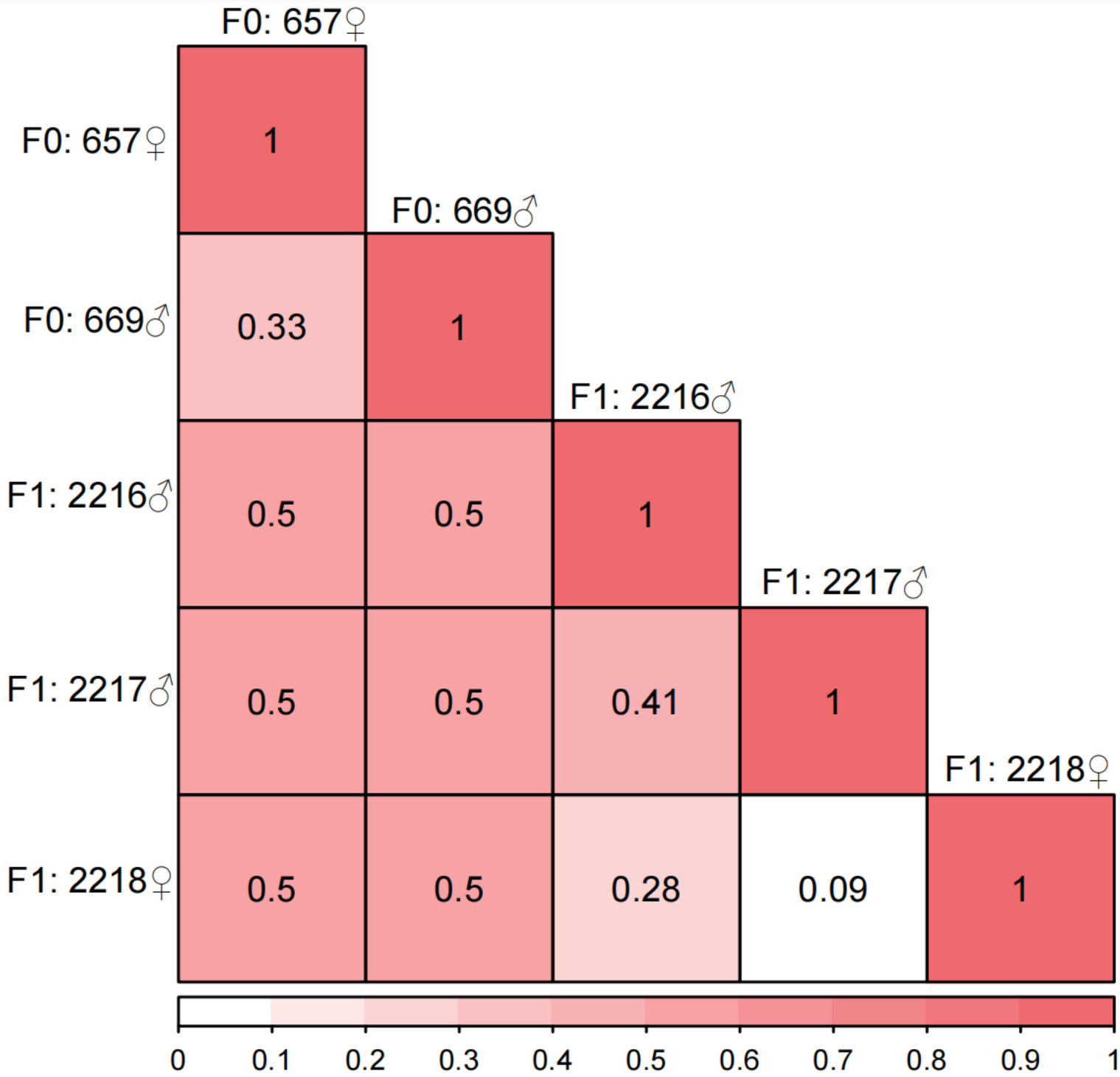

Supplement: qzaf071_Supplementary_Data [file qzaf071_supplementary_data.zip › Figure S15.pdf]

A

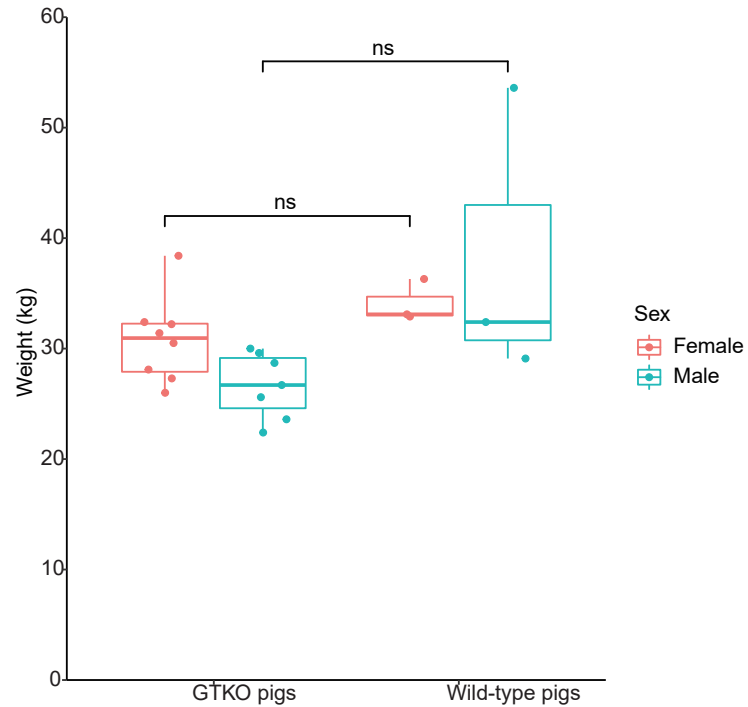

B

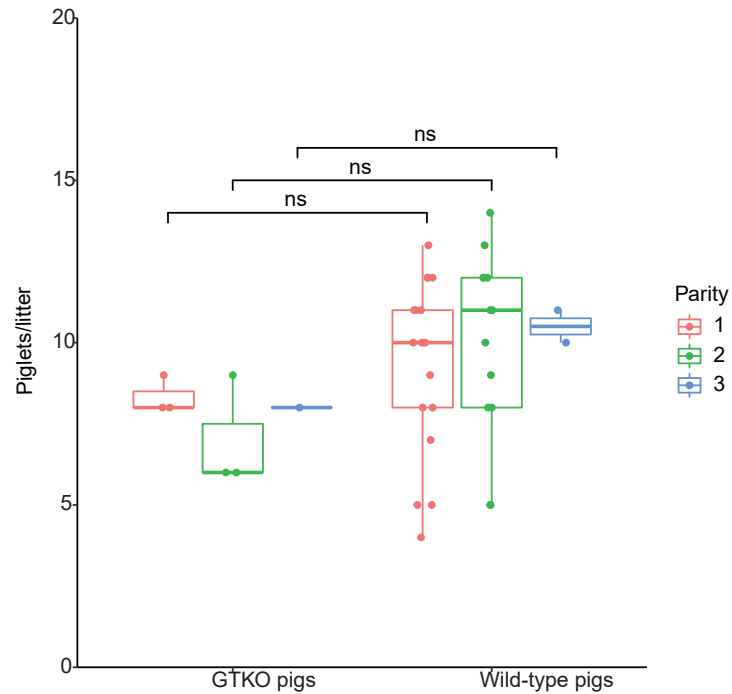

Supplement: qzaf071_Supplementary_Data [file qzaf071_supplementary_data.zip › Figure S1.pdf]

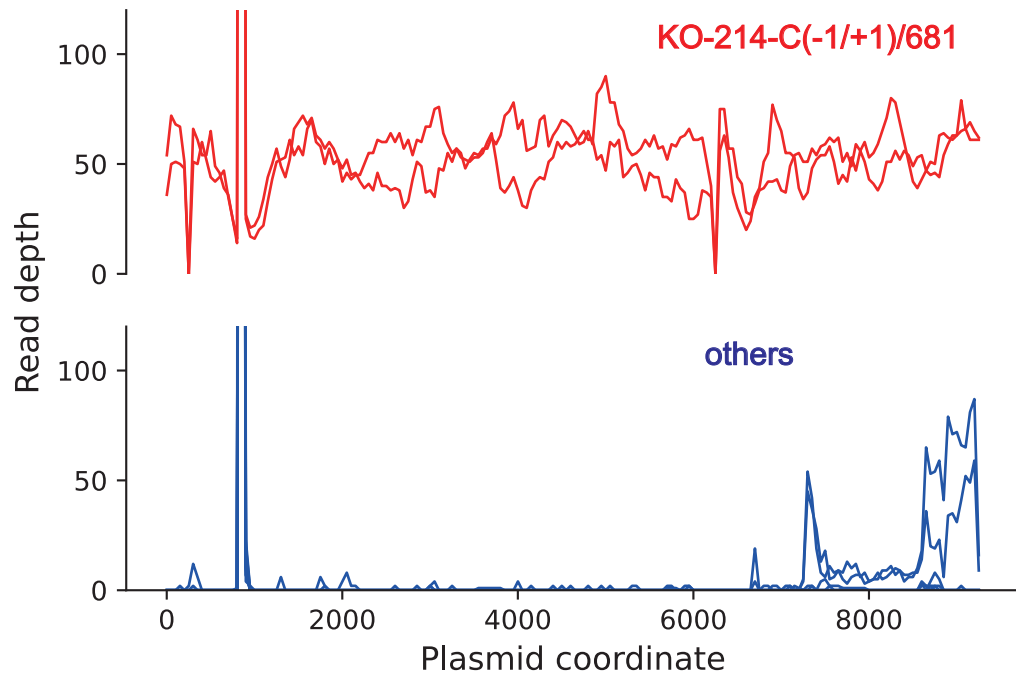

Supplement: qzaf071_Supplementary_Data [file qzaf071_supplementary_data.zip › Figure S2.pdf]

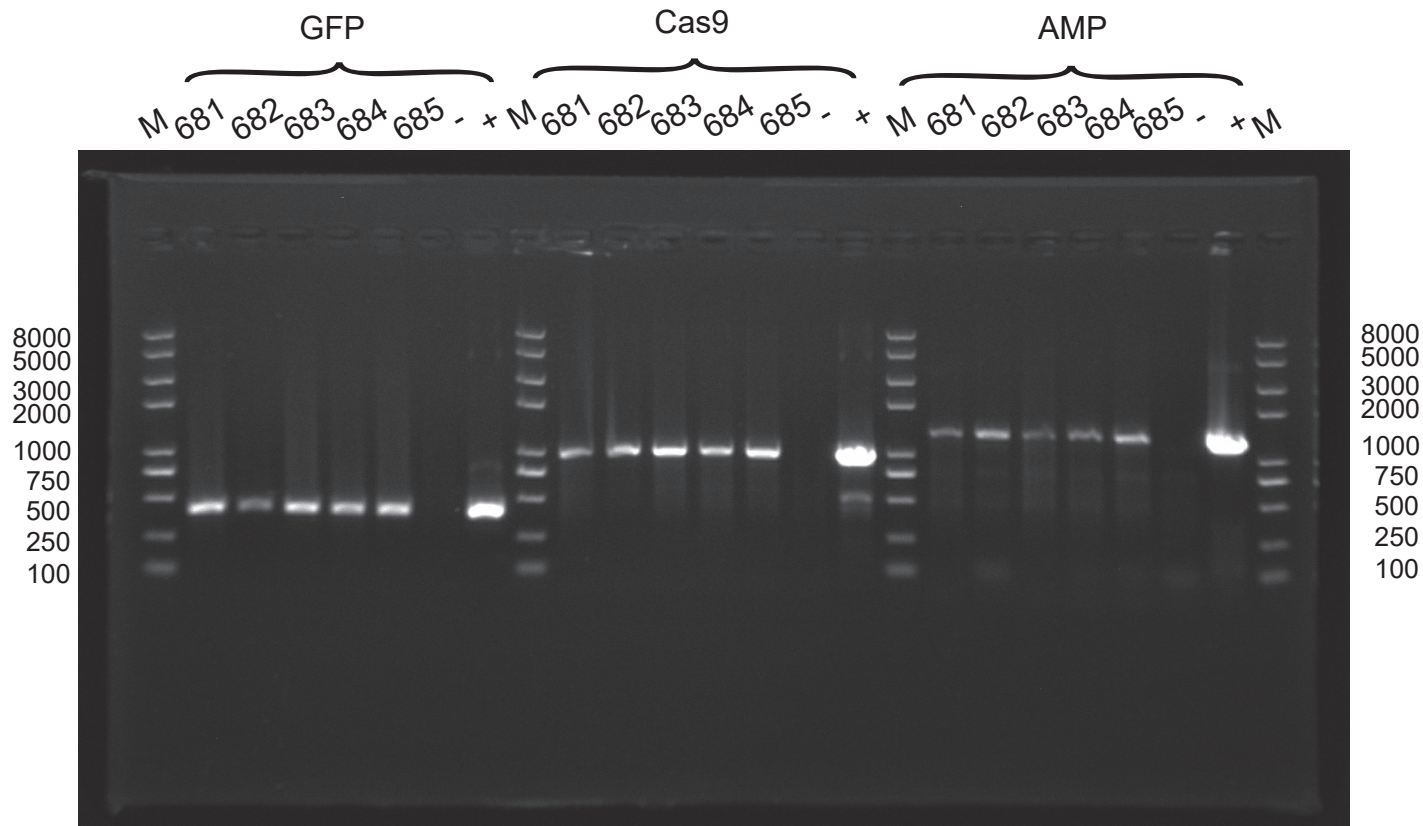

Supplement: qzaf071_Supplementary_Data [file qzaf071_supplementary_data.zip › Figure S3.pdf]
